# Supplementary material for: Macrophage manufacturing and engineering with 5′-Cap1 and N1-methylpseudouridine-modified mRNA
Source: Mol Ther Methods Clin Dev. 2024 Jul 31;32(3):101307. doi: 10.1016/j.omtm.2024.101307 (PMC11369376; doi:10.1016/j.omtm.2024.101307)
Supplement: Document S1. Figures S1–S4 and Table S1 [file mmc1.pdf]

OMTM, Volume *32*

Supplemental information

Macrophage manufacturing and engineering with 5′-Cap1 and N1-methylpseudouridine-modified mRNA

Peixuan Zhang, Yantai Wang, Jinfeng Jiang, Chao Yang, Xianxia Liu, Tingjun Lei, Xiangjun Meng, Jihong Yang, Ping Ding, Jie Chen, and Qintong Li

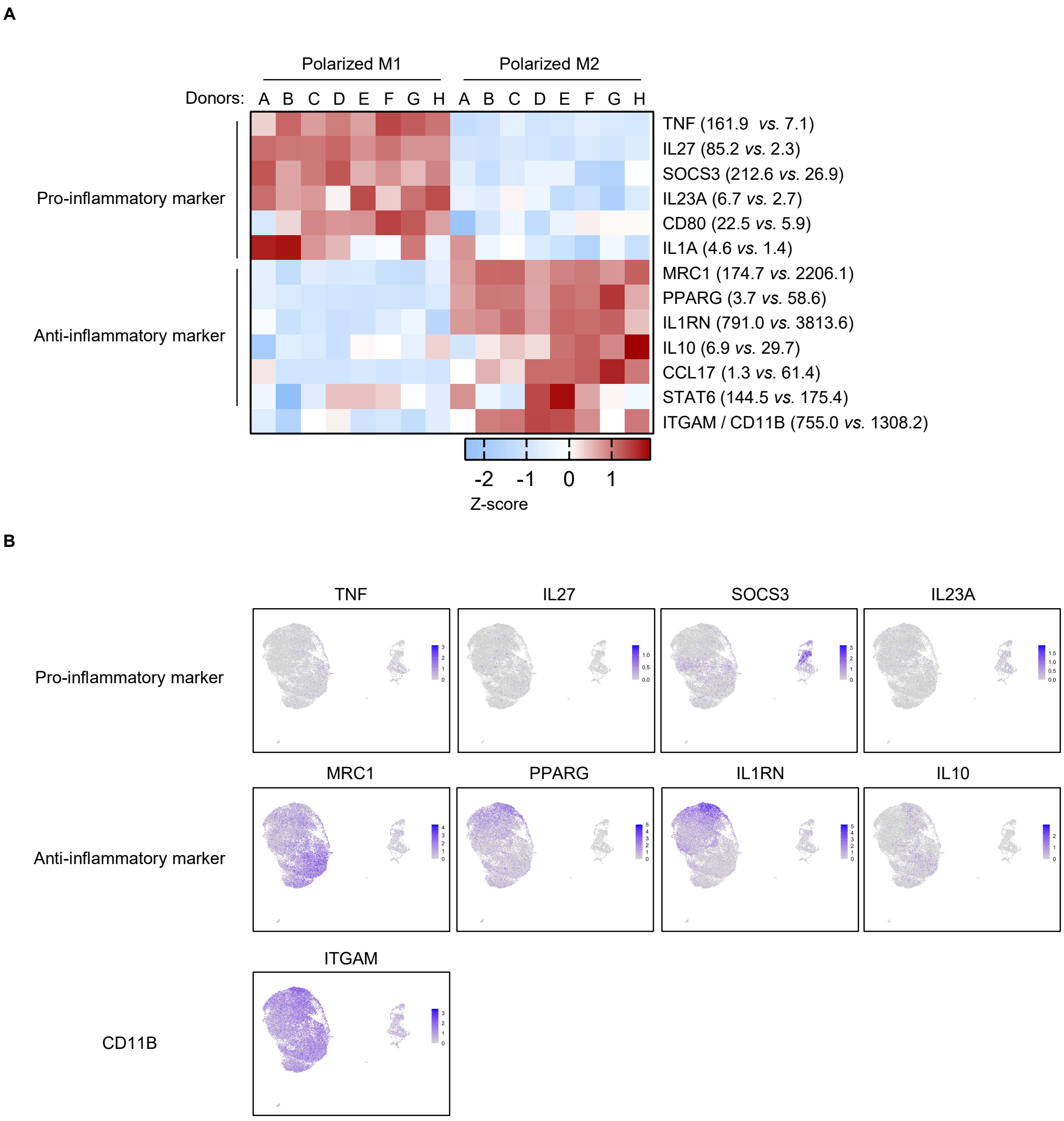

Figure S1. Selection of anti-inflammatory and pro-inflammatory marker genes by comparing paired unpolarized, M1 and M2 macrophages, and their expression pattern at single-cell resolution

(A) Heatmap presentation of inter-personal variation of frequently used inflammatory (TNF, IL27, SOCS3, IL23A, CD80 and IL1A) and anti-inflammatory (MRC1, PPARG, IL1RN, IL10, CCL17 and STAT6) markers in the published literature. Unpolarized macrophages were derived from 8 donor materials (Donors A-H), and polarized individually into M1 (induced by IFN-γ) and M2 state (induced by IL-4), respectively. In total, 24 RNA-seq experiments were performed (M1, unpolarized and M2 for each donor), and differential gene expression was analyzed using paired M1 and M2 macrophages from each donor. Of note, only TNF, IL27, SOCS3 and IL23A (but not CD80 or IL1A) are expressed at higher levels in M1 derived from any given donor than M2 derived from all 8 donors. Similarly, regarding anti-inflammatory markers, MRC1, PPARG and IL1RN are constantly higher in M2 than M1, while other markers are less robust.

(B) UMAP visualization of the expression pattern of TNF, IL27, SOCS3 as well as IL23A (inflammatory markers), and MRC1, PPARG, IL1RN and IL10 (anti-inflammatory markers) as well as CD11B in our single-cell sequencing dataset (Figure 3).

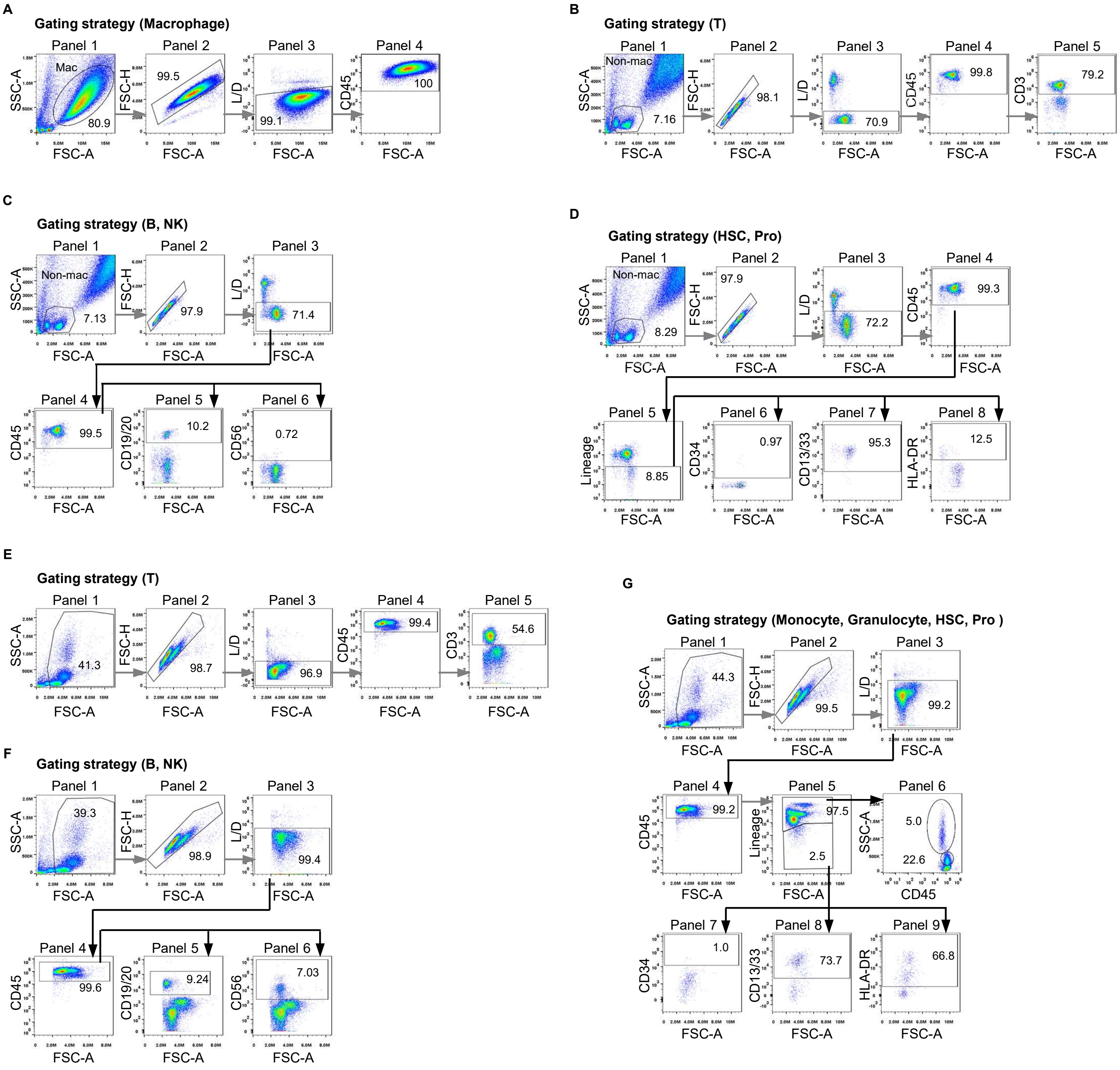
A
Gating strategy (Macrophage)
Panel 1
Panel 2
Panel 3
Panel 4
Mac
80.9
99.5
99.1
100
SSC-A
FSC-H
L/D
CD45
FSC-A
B
Gating strategy (T)
Non-mac
7.16
98.1
70.9
99.8
79.2
CD3
C
Gating strategy (B, NK)
7.13
97.9
71.4
99.5
10.2
0.72
CD19/20
CD56
D
Gating strategy (HSC, Pro)
8.29
97.9
72.2
99.3
8.85
0.97
95.3
12.5
Lineage
CD34
CD13/33
HLA-DR
E
Gating strategy (T)
41.3
98.7
96.9
99.4
54.6
F
Gating strategy (B, NK)
39.3
98.9
99.4
99.6
9.24
7.03
G
Gating strategy (Monocyte, Granulocyte, HSC, Pro)
44.3
99.5
99.2
99.2
97.5
2.5
5.0
22.6
1.0
73.7
66.8

Figure S2. Gating strategies for various cell types

(A) Gating strategy of FACS analyses to quantify macrophages in Mo-Mac. Cells were gated based on side scatter area (SSC-A) and forward scatter area (FSC-A) (panel 1), then gated sequentially to include singlet cells (panel 2), live cells (panel 3) and $CD45^+$ cells (panel 4).

(B) Gating strategy to quantitate T cells in Mo-Mac. Non-macrophage cells (Non-mac) were gated based on side scatter area (SSC-A) and forward scatter area (FSC-A) (panel 1), then sequentially gated to include singlet cells (panel 2), live cells (panel 3) and $CD45^+$ cells (panel 4). $CD45^+$ cells were further gated to identify $CD3^+$ T cells (panel 5).

(C) Gating strategy to quantify B and natural killer (NK) cells in Mo-Mac. Non-macrophage cells (Non-mac) were gated based on side scatter area (SSC-A) and forward scatter area (FSC-A) (panel 1), then sequentially gated to include singlet cells (panel 2), live cells (panel 3) and $CD45^+$ cells (panel 4). $CD45^+$ cells were further gated to identify $CD19^+CD20^+$ B cells (panel 5) and $CD56^+$ NK cells (panel 6).

(D) Gating strategy to quantify hemopoietic stem cells (HSC) and promyelocytes/promonocytes (Pro) in Mo-Mac. Non-macrophage cells (Non-mac) were gated based on side scatter area (SSC-A) and forward scatter area (FSC-A) (panel 1), then sequentially gated to include singlet cells (panel 2), live cells (panel 3) and $CD45^+$ cells (panel 4). $CD45^+$ cells were gated for lineage-negative cells ($CD3^-$, $CD16^-$, $CD19^-$, $CD20^-$, $CD56^-$ and $CD14^-$) (panel 5). Lineage-negative cells were then gated to identify $CD34^+$ HSC (panel 6), and $CD13^+CD33^+$ promyelocytes or HLA-DR^+ promonocytes (panel 7 and 8).

(E) Gating strategy to quantify T cells in elutriation and leukapheresis materials. Cells were gated based on side scatter area (SSC-A) and forward scatter area (FSC-A) (panel 1), then gated sequentially to include singlet cells (panel 2), live cells (panel 3) and $CD45^+$ cells (panel 4). $CD45^+$ cells were further gated to identify $CD3^+$ T cells (panel 5).

(F) Gating strategy to quantify B and natural killer (NK) cells in elutriation and leukapheresis materials. Cells were gated based on side scatter area (SSC-A) and forward scatter area (FSC-A) (panel 1), then gated sequentially to include singlet cells (panel 2), live cells (panel 3) and $CD45^+$ cells (panel 4). $CD45^+$ cells were further gated to identify $CD19^+CD20^+$ B cells (panel 5) and $CD56^+$ NK cells (panel 6).

(G) Gating strategy to quantify monocytes, granulocytes, hematopoietic stem cells (HSC) and promyelocytes/promonocytes (Pro) in elutriation and leukapheresis materials. Cells were gated based on side scatter area (SSC-A) and forward scatter area (FSC-A) (panel 1), then gated sequentially to include singlet cells (panel 2), live cells (panel 3) and $CD45^+$ cells (panel 4). $CD45^+$ cells were gated for lineage-negative cells ($CD3^-$, $CD16^-$, $CD19^-$, $CD20^-$, $CD56^-$ and $CD14^-$) (panel 5). Lineage-negative cells were then gated to identify $CD34^+$ HSC (panel 7), and $CD13^+CD33^+$ promyelocytes or HLA-DR^+ promonocytes (panel 8 and 9). Lineage-positive cells were then gated to identify $CD45^{low}$ granulocyte (panel 6) and $CD45^{high}$ monocyte (panel 6).

A

B

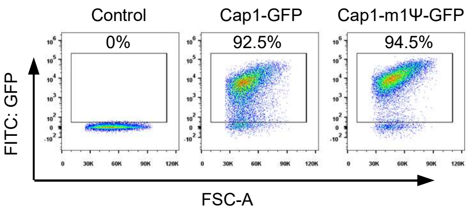

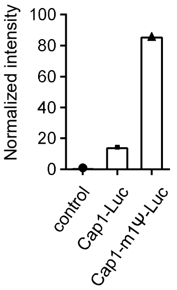

Figure S3. Verification of the expression of Cap1 and Cap1-m1Ψ mRNA

(A) Flow cytometry analysis of the expression of Cap1-GFP mRNA and Cap1-m1Ψ-GFP mRNA in electroporated macrophages.

(B) Bioluminescence-based detection of the expression of Cap1-Luc mRNA and Cap1-m1Ψ-Luc mRNA in electroporated macrophages.

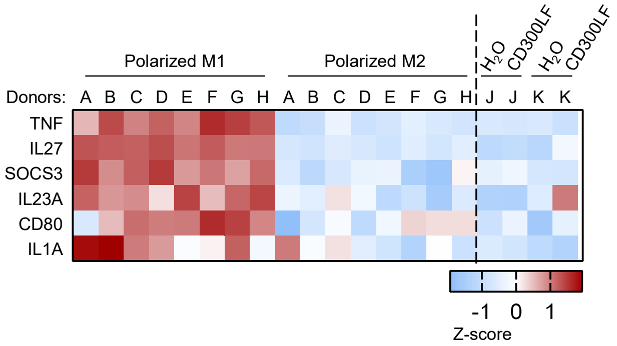

Figure S4. Cap1-m1Ψ-CD300LF mRNA-engineered macrophages are not in an inflammatory state

Heatmap showing the expression level of pro-inflammatory genes (TNF, IL27, SOCS3, IL23A) in Cap1-m1Ψ-CD300LF mRNA-engineered macrophages, M1- or M2-polarized macrophages. Considering the intra-personal variations, only constantly upregulated genes in M1-macrophages were selected as pro-inflammatory markers (Figure S1).

Table S1. Top20 differentially expressed genes induced by unmodified mRNA and attenuated by m1Ψ-modified mRNA

gene_name	Upregulated by unmodified mRNA (fold change)		gene_name	Downregulated by unmodified mRNA (fold change)	
	Cap1 mRNA vs. Control	Cap1-m1Ψ mRNA vs. Control		Cap1 mRNA vs. Control	Cap1-m1Ψ mRNA vs. Control
CXCL11	1002.5	12.4	JAML	-32.8	-1.6
CXCL10	922.3	10.8	FABP4	-22.7	-1.3
ACOD1	540.3	91.7	LINC01503	-22.1	-2.1
IDO1	519.0	44.0	AL844908.1	-19.7	-1.3
CCL8	420.0	9.0	AC078850.1	-18.1	-1.0
AC007991.2	367.2	26.2	DOK2	-17.3	-1.3
APOBEC3A	282.3	20.1	SERPINE1	-14.7	-1.2
TNFAIP6	263.2	4.9	MACROD1	-14.6	-1.7
CD38	210.7	11.0	AL031123.1	-14.5	-1.7
CALHM6	203.1	9.5	SNAI3	-14.3	-2.4
GBP1P1	163.4	15.8	LPL	-13.9	-2.0
PDGFRL	135.5	9.5	LINC01010	-13.7	-1.5
GJA4	134.9	31.8	DBP	-13.3	-1.5
IL27	126.2	1.8	RASAL1	-12.4	-1.5
NEURL3	119.9	5.1	SIGLEC7	-12.0	-1.5
IGFBP4	107.9	4.6	ASGR1	-12.0	-1.5
IFITM1	103.3	11.3	CD300LB	-11.5	-1.2
ZBP1	90.0	6.1	PC	-10.9	-1.4
TNFSF10	85.6	7.0	TM4SF19	-10.8	-1.8
LAG3	82.9	7.9	DPCD	-10.7	-1.4
