# Supplementary material for: Macrophage manufacturing and engineering with 5′-Cap1 and N1-methylpseudouridine-modified mRNA
Source: Mol Ther Methods Clin Dev. 2024 Jul 31;32(3):101307. doi: 10.1016/j.omtm.2024.101307 (PMC11369376; doi:10.1016/j.omtm.2024.101307)
Supplement: Document S2. Article plus supplemental information [file mmc2.pdf]

Molecular Therapy
Methods & Clinical Development
Original Article

Macrophage manufacturing and engineering with 5′-Cap1 and N1-methylpseudouridine-modified mRNA

Peixuan Zhang,[1,5] Yantai Wang,[2,5] Jinfeng Jiang,[3] Chao Yang,[1] Xianxia Liu,[4] Tingjun Lei,[4] Xiangjun Meng,[4] Jihong Yang,[4] Ping Ding,[3] Jie Chen,[2] and Qintong Li[1]

[1]Departments of Obstetrics & Gynecology and Pediatrics, West China Second University Hospital, Key Laboratory of Birth Defects and Related Diseases of Women and Children, Ministry of Education, Development and Related Diseases of Women and Children Key Laboratory of Sichuan Province, Center of Growth, Metabolism and Aging, State Key Laboratory of Biotherapy and Collaborative Innovation Center of Biotherapy, Sichuan University, Chengdu 610041, Sichuan, China; [2]Department of General Surgery, Breast Center, West China Hospital, Sichuan University, Chengdu 610041, Sichuan, China; [3]Non-coding RNA and Drug Discovery Key Laboratory of Sichuan Province, Chengdu Medical College, Chengdu 610500, Sichuan, China; [4]Division of Cell Manufacturing, Sichuan Cunde Therapeutics, Chengdu 610093, Sichuan, China

Macrophage-based cell therapeutics is an emerging modality to treat cancer and repair tissue damage. A reproducible manufacturing and engineering process is central to fulfilling their therapeutic potential. Here, we establish a robust macrophage-manufacturing platform (Mo-Mac) and demonstrate that macrophage functionality can be enhanced by N1-methylpseudouridine (m1Ψ)-modified mRNA. Using single-cell transcriptomic analysis as an unbiased approach, we found that >90% cells in the final product were macrophages while the rest primarily comprised T cells, B cells, natural killer cells, promyelocytes, promonocytes, and hematopoietic stem cells. This analysis also guided the development of flow-cytometry strategies to assess cell compositions in the manufactured product to meet requirements by the National Medical Products Administration. To modulate macrophage functionality, as an illustrative example we examined whether the engulfment capability of macrophages could be enhanced by mRNA technology. We found that efferocytosis was increased *in vitro* when macrophages were electroporated with m1Ψ-modified mRNA encoding CD300LF (CD300LF-mRNA-macrophage). Consistently, in a mouse model of acute liver failure, CD300LF-mRNA-macrophages facilitated organ recovery from acetaminophen-induced hepatotoxicity. These results demonstrate a GMP-compliant macrophage-manufacturing process and indicate that macrophages can be engineered by versatile mRNA technology to achieve therapeutic goals.

INTRODUCTION

Macrophages are multifunctional innate immune cells essential for pathogen defense, tissue development, and homeostatic maintenance.[1] Macrophages reside in almost all organs and are produced via two routes.[2] During early embryogenesis, erythromyeloid progenitor cells from yolk sac seed and differentiate within developing organs to generate resident tissue macrophages (RTMs).[3,4] In addition, monocytes originating from the bone marrow can also differentiate into macrophages. As a result, most adult organs are replete with both RTMs and monocyte-derived macrophages (Mo-macs).[2] Regardless of their developmental origins, both RTMs and Mo-macs share core functionalities as sentinel cells to engulf pathogens, defected and dying cells, and metabolic waste.[5] Under pathological conditions, peripheral monocytes are recruited to the subtissular lesions to generate Mo-macs, functionally compensating damaged tissue-resident macrophages.[6]

Macrophages are highly responsive to cues within the tissue environment to adopt diverse phenotypes and functionalities.[2] Colony-stimulating factor 1 (CSF1) is a cytokine essential for macrophage differentiation and maintenance *in vivo*[5] and is frequently used to induce the differentiation of human monocytes into macrophages *in vitro*.[7] CSF1-induced human macrophages, or M(CSF1), exhibit immunosuppressive properties.[8] Like their counterparts *in vivo*, M(CSF1) can adopt proinflammatory phenotypes (M1) *in vitro* when treated with interferon-γ (IFN-γ) and tissue-reparative phenotypes (M2)

Received 16 September 2023; accepted 26 July 2024;
https://doi.org/10.1016/j.omtm.2024.101307.

[5]These authors contributed equally

Correspondence: Ping Ding, Non-coding RNA and Drug Discovery Key Laboratory of Sichuan Province, Chengdu Medical College, Chengdu 610500, Sichuan, China.
E-mail: dingping@cmc.edu.cn
Correspondence: Jie Chen, Department of General Surgery, Breast Center, West China Hospital, Sichuan University, Chengdu 610041, Sichuan, China.
E-mail: chenjiewestchina@163.com
Correspondence: Qintong Li, Departments of Obstetrics & Gynecology and Pediatrics, West China Second University Hospital, Key Laboratory of Birth Defects and Related Diseases of Women and Children, Ministry of Education, Development and Related Diseases of Women and Children Key Laboratory of Sichuan Province, Center of Growth, Metabolism and Aging, State Key Laboratory of Biotherapy and Collaborative Innovation Center of Biotherapy, Sichuan University, Chengdu 610041, Sichuan, China.
E-mail: liqintong@scu.edu.cn

© 2024 The Author(s).
Published by Elsevier Inc. on behalf of The American Society of Gene and Cell Therapy.
This is an open access article under the CC BY license (http://creativecommons.org/licenses/by/4.0/).

when treated with interleukin-4 (IL-4).[9] Beyond the classical M1/M2 classification, macrophages respond to diverse signals to adopt M1-like, M2-like, or hybrid phenotypes and functionalities in their subtissular niches.[10]

Macrophages are attractive therapeutic targets and vessels in the modulation of disease progression. For example, tumor-associated macrophages (TAMs) are thought to adopt protumoral functions.[11] Various antibodies and small molecules have been developed to directly eliminate TAMs or block the recruitment of peripheral monocytes to produce TAMs.[12] However, these approaches are largely ineffective in clinical trials because the functions of TAMs are highly complex and heterogeneous.[13,14] Alternatively, macrophage-based cell therapeutics are emerging as a novel modality to treat cancer and repair tissue damage.[15] Macrophages can be manufactured from peripheral monocytes collected by leukapheresis. A recent study demonstrated that macrophages transduced with chimeric antigen receptor (CAR-M) enables elimination of solid tumors by adopting proinflammatory M1 phenotype to boost the anti-tumor microenvironment.[16] In addition, macrophage-based therapy is also effective in repairing tissue damage.[17]

To manufacture macrophages on an industrial scale, leukapheresis materials are commonly used as starting materials. Subsequently, two methods are usually utilized to further enrich monocytes. One way is to enrich monocytes by elutriation and the other is to enrich monocytes by anti-CD14 affinity purification. Both methodologies are used in the manufacturing industry to generate monocyte-derived macrophages. Elutriation is less costly than affinity purification and is based on centrifugal forces to separate monocytes from other immune cells. Affinity purification with anti-CD14 antibody-coated beads offers higher purity of monocytes (∼95%) than elutriation (>70%). By comparison, to manufacture T cell product it is common practice to use antibody-coated beads to purify T cells, followed by amplification of T cells by several orders of magnitude. Thus, antibody-coated beads are much diluted in the final T cell product and safe for infusion. However, monocytes are non-proliferative cell types, and a much larger quantity of antibody-coated beads are required, eliciting a much higher manufacturing cost.

The highly malleable nature of macrophages presents both opportunities and challenges for manufacturing cell therapy. Because macrophages are highly responsive to surrounding cues, variations on various manufacturing steps may impact macrophage identity, phenotype, and functionality. These variations in the manufacture of cell therapies are largely understudied and present a major challenge for the interpretation of clinical trial results.[18] To minimize variations in manufacturing, a single centralized facility is arguably the best way to ensure product reproducibility and quality. In addition, the tools used to engineer macrophages can have potent biological effects. Adenoviral vector for CAR-M engineering imparts a sustained proinflammatory M1 phenotype, favoring anti-tumor immunity. However, for tissue regeneration, proinflammatory adenoviral vector would be unsuitable because M2-like phenotypes and functionalities are desired. Thus, the biological effects of engineering tools on macrophages need to be carefully investigated.

We recently developed a platform to generate good manufacturing practice (GMP)-grade therapeutic macrophages in compliance with the guidelines from the National Medical Products Administration (NMPA), China. For regulatory considerations, it is important to define the cell-type compositions to address safety concerns but also to ensure reproducible, high-quality manufacture of the intended cell type. Here, we demonstrate a reproducible manufacture of high-quality macrophage product (Mo-Mac). To address regulatory concerns on cell-type heterogeneity in the final product, we used single-cell transcriptomic sequencing as an unbiased approach to characterize cell-type compositions in Mo-Mac and to guide the development of a flow-cytometry-based method as quality assurance and control steps in the manufacturing process. Lastly, we show that the phagocytic ability of manufactured macrophages can be enhanced by versatile mRNA technology to achieve therapeutic goals.

RESULTS

Overview of the Mo-Mac manufacturing process

An overview of the Mo-Mac manufacturing process is presented in Figure 1A. To develop macrophage-based therapies, the manufacturing process was optimized using peripheral blood monocytes collected by leukapheresis and enriched by subsequent elutriation (day 1). Enriched monocytes were differentiated in culture bags to generate macrophages (days 2–8). Harvested macrophages were engineered by electroporation of *in vitro* transcribed mRNA encoding therapeutic proteins of interest. Various quality assurance and control analyses as well as microbiological examinations (days 9–28) were carried out in accordance with NMPA guidelines (Figure 1A).

To develop a robust manufacturing process to enrich monocytes, we carried out 23 test runs. Monocytes in peripheral blood from 23 healthy volunteers were collected by leukapheresis using the Spectra Optia apheresis system. The percentage of monocytes ranged from 11.5% to 32.1% (median, 21.0%; mean, 22.6% ± 1.1%), quantified by an ABX Pentra 60 hematology analyzer (Figure 1B). Monocytes collected by leukapheresis were further purified using the Elutra system, resulting in a median of 73.5% monocytes (range, 62.6%–91.0%; mean, 75.2% ± 2.0%) (Figure 1C). The identity and purity of monocytes was further verified by flow cytometry ($CD14^+$) (Figures 1D and 1E). The elutriation step reproducibly enriched monocytes (Figure 1F). The recovery rate after leukapheresis and elutriation was similar to those from our previous 85 test runs to develop a dendritic-cell manufacturing platform.[19] In most circumstances, monocytes were enriched in fraction 4, 5, or 6 (Figure 1G). Due to variations in donor materials, monocytes from single fraction or combined fractions were used to ensure that at least 10^9 monocytes were cultured. Of 23 batches produced, 16 were manufactured using faction 6 alone, five from combined fractions 5 and 6, and two from combined fractions 4, 5, and 6 (Figure 1H).

NMPA guidelines recommend using multiple parameters to characterize the viability and functional properties of the manufactured

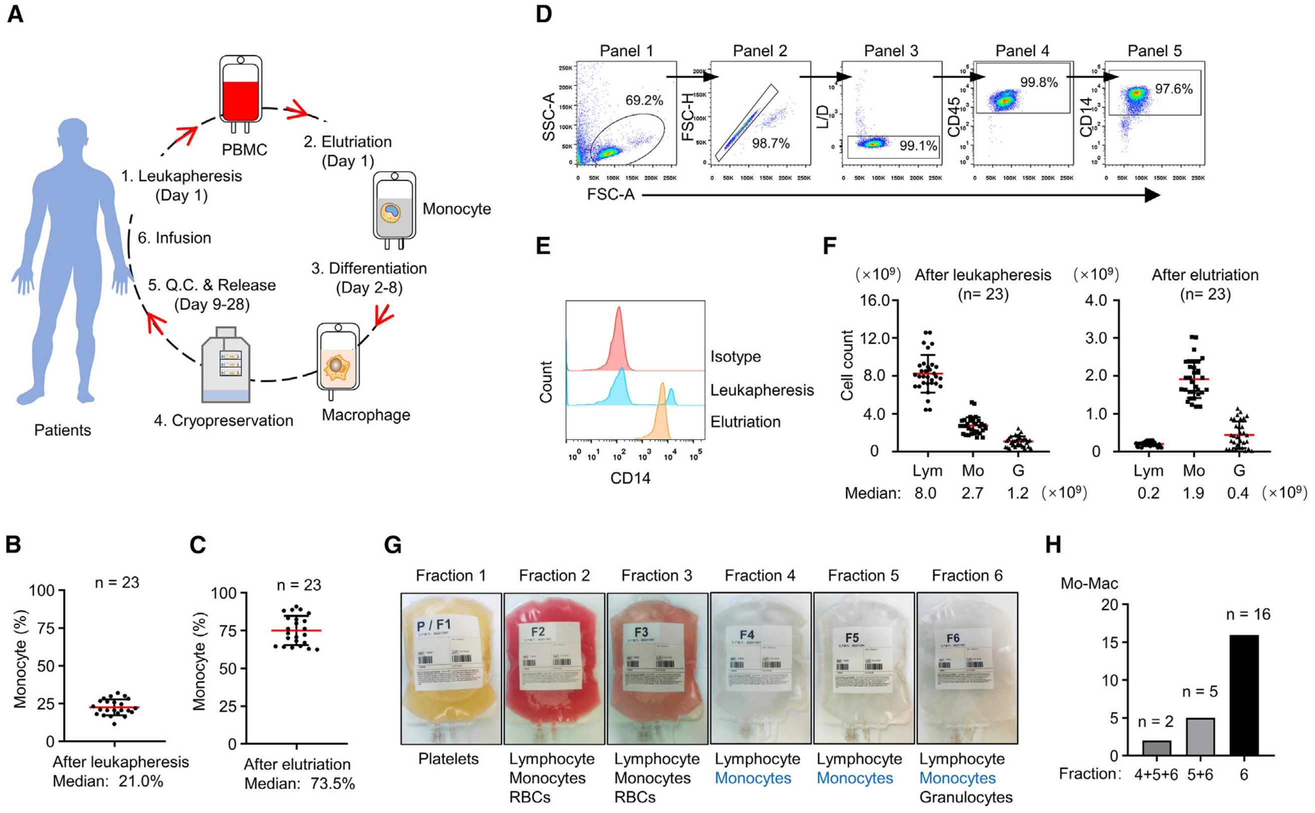

Figure 1. Overview of GMP-grade macrophage-manufacturing process

(A) An overview of the manufacturing platform for GMP-grade macrophages (Mo-Mac). Peripheral blood monocytes are enriched by leukapheresis and elutriation (day 1) and differentiated into macrophages (days 2–8). The Mo-Mac product, containing >90% macrophages, is stored in liquid nitrogen until the day of infusion. For various quality control steps (days 9–28), a portion of stored cells is revived. (B) Percentage of monocytes in leukapheresis materials from 23 healthy donors determined by ABX Pentra 60 hematology analyzer. (C) Percentage of monocytes in elutriation materials from 23 healthy donors determined by ABX Pentra 60 hematology analyzer. (D) Flow cytometry gating strategy to analyze the percentage of monocytes after leukapheresis and elutriation. Cells were gated based on side scatter area (SSC-A) and forward scatter area (FSC-A) (panel 1), then gated to include singlet cells (panel 2) and live cells (panel 3). $CD45^+$ cells (panel 4) were further gated to identify $CD14^+$ monocytes (panel 5). (E) Representative result of flow-cytometry analysis of monocyte purity after leukapheresis and elutriation. Isotype was used as a negative control to determine the non-specific background of CD14. (F) Quantities of lymphocytes (Lym), monocytes (Mo), and granulocytes (G) in leukapheresis and elutriation materials from 23 healthy donors. (G) Representative example of fractions separated by elutriation. Cell compositions in each fraction were determined by ABX Pentra 60 hematology analyzer, and enriched components are shown for each fraction. (H) Statistics of elutriation fractions used to manufacture Mo-Mac from 23 donor materials. Because of donor variations, monocytes from single or combined fractions were used.

cell product. Several quality control steps were carried out to ensure the quality of manufactured macrophages. Microscopic inspection revealed that most cells were round and had a number of short protrusions of the cytoplasm, characteristic of macrophage morphology (Figure 2A). A monoclonal antibody, 25F9, is widely used to identify macrophages because it recognizes an antigen specifically expressed in monocyte-derived macrophages.[20] In addition, macrophages express CD14 and CD206. Immunophenotyping by flow-cytometry analysis was carried out to verify the identity and phenotype of macrophages (Figure 2B). In 23 manufactured batches, the median percentage was 95.3% for $25F9^+$ (range 71.3%–99.6%, mean 92.4% ± 1.6%) (Figure 2C, left panel), and 93.3% for $CD206^+$ (range 79.9%–99.8%, mean 93.5% ± 1.0%) (Figure 2C, right panel). To measure the phagocytic activity, macrophages were incubated with pHrodo *E. coli* BioParticles for 1 h. The median percentage of fluorescence-positive macrophages was 63.0% (range 39.3%–95.9%, mean 66.7% ± 3.3%) (Figure 2D). The median cellular viability was 94.1% (range 92.1%–99.8%, mean 95.6% ± 0.6%) (Figure 2E). The median yield of macrophages per monocyte was 65.1% (range 47.0%–82.3%, mean 66.5% ± 2.1%) (Figure 2F). Among 23 donors, the macrophage yield was positively proportional to the quantity of monocytes at the beginning of the culturing process (Figure 2G). Typically, more than 5×10^8 macrophages could be manufactured from a single leukapheresis collection.

Single-cell transcriptomic analysis of Mo-Mac cell-type compositions

NMPA guidelines require the identification of the cell compositions in the manufactured cell product. To dissect cell-type compositions in an unbiased manner, we carried out single-cell transcriptomic

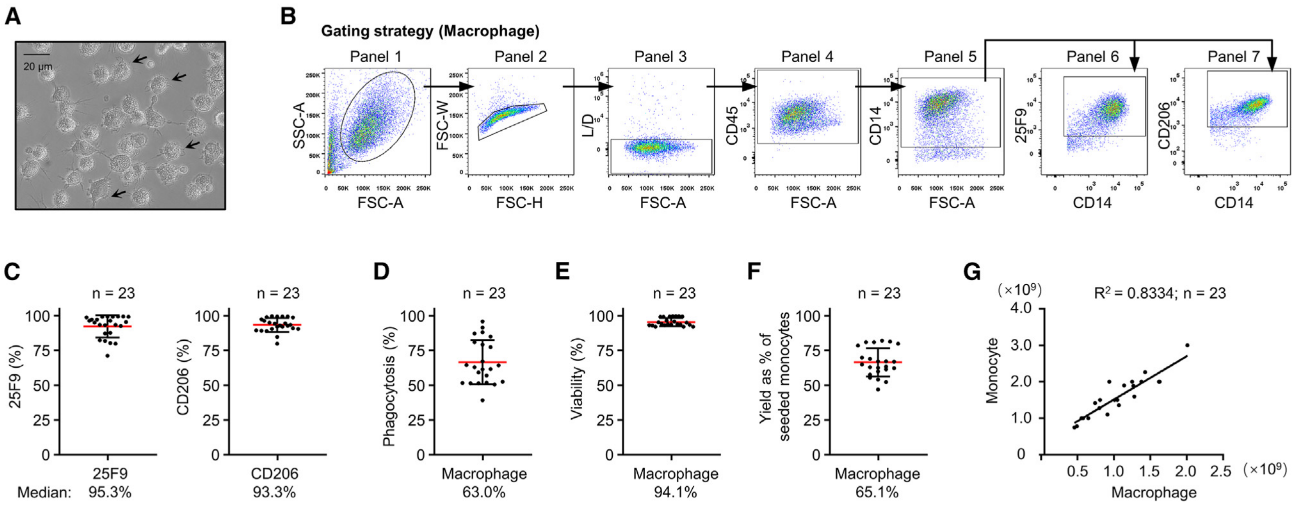

Figure 2. Reproducible production of GMP-grade Mo-Mac

(A) Microscopic examination of Mo-Mac product. Cells were harvested from the culture bag and replated on a six-well plate for microscopic examination of cell morphology. Macrophages are denoted with an arrow. (B) Phenotyping of Mo-Mac by flow-cytometry analysis. Macrophages were gated based on side scatter area (SSC-A) and forward scatter area (FSC-A) (panel 1), then gated to include singlet cells (panel 2) and live cells (panel 3). $CD45^+$ cells (panel 4) were further gated to identify $CD14^+$ cells (panel 5). Macrophage phenotypic markers $25F9^+$ (panel 6) and $CD206^+$ (panel 7) were gated from $CD14^+$ cells. (C) Phenotyping of 23 batches of Mo-Mac product using flow cytometry gating strategies in (B). (D) Phagocytic ability of macrophages from 23 batches of Mo-Mac product. The percentage of live cells containing pHrodo *E. coli* BioParticles was determined by flow-cytometry analysis. (E) Viability of macrophages from 23 batches of Mo-Mac product.
(F) Yield of macrophages from 23 batches of Mo-Mac product. (G) Correlation analysis between the quantity of seeding monocytes and the number of generated macrophages from 23 batches of Mo-Mac product.

analysis of Mo-Mac products from two donors. 12,212 and 9,509 cells were profiled using droplet-based single-cell RNA sequencing (RNA-seq) (10× Genomics Chromium) for donor 1 and donor 2, respectively. After quality control, high-quality sequencing data were obtained containing 8,756 and 7,545 cells for donor 1 and donor 2, respectively. All cells were integrated and subjected to donor correction by Harmony.[21] Five distinct clusters were identified by unsupervised clustering using the Louvain method.[22] Cell types were first calculated using the SingleR package,[23] assigned with the most differentially expressed marker genes using Seurat,[24] and annotated by lineage-specific canonical markers. These analyses generated five distinct cell groups, and the top 20 representative marker genes using *Z*-score calculation are illustrated in a heatmap for non-macrophage cell types (Figure 3A). For a total of 323 bystander cells combined from two donors, the expression levels of the top five marker genes per bystander cell type are illustrated in Figure 3B. Uniform manifold approximation and projection (UMAP) visualization of these cells revealed that the majority of cells were identified as macrophages (97.96% and 98.09% for donors 1 and 2, respectively) (Figure 3C). The percentage of remaining immune cell types included 0.38% and 0.66% T/natural killer (NK) cells, 0.24% and 0.98% B cells, 0.96% and 0.04% promyelocytes/promonocytes, and 0.47% and 0.23% hematopoietic stem cells (HSCs) for donors 1 and 2, respectively (Figure 3C).

To assess the reproducibility of the single-cell transcriptomic profiling, we repeated the analysis using Mo-Mac product derived from another donor (donor 3). We intentionally selected donor 3 because of the presence of excessive T lymphocytes in the elutriation material (donor 1: 0.39%; donor 2: 0.68%; donor 3: 34.76%). Thus, we could ask whether excessive T cells in the starting material would compromise the quality of manufactured macrophage, and whether our bioinformatics analysis pipeline was robust enough to detect bystander cells with various quantities. The same analyses as described for donors 1 and 2 were carried out for donor 3. After quality control, high-quality sequencing data were obtained containing 4,617 cells. UMAP visualization of Mo-Mac product derived from donor 3 showed the composition of 34.76% T/NK cells, 0.17% B cells, 0.09% promyelocytes/promonocytes, and 0.06% HSCs (Figure 3D). Harmony integration demonstrated that samples from these three donors were well mixed and grouped by defined cell types (Figure 3E). We analyzed macrophage markers CD68, CD163, and CD206, which were highly expressed in the macrophage population by single-cell analysis. In contrast, CD3D expression was restricted to the T cell population as expected (Figure 3F). These results demonstrated that our bioinformatics pipelines are robust enough to catalog cell types in various quantities, but also that excessive T cells did not interfere with the manufacturing process because the cellular state of macrophages from these donors were indistinguishable. However, excessive T cells seemed to survive from the culturing process and end up in the final product. Donor 3 was the only case we had so far with excessive T cells present in the elutriation product. Of note, we do not recommend infusing product with excessive T cells, due to the present uncertainty regarding safety. In the case of when the second round of

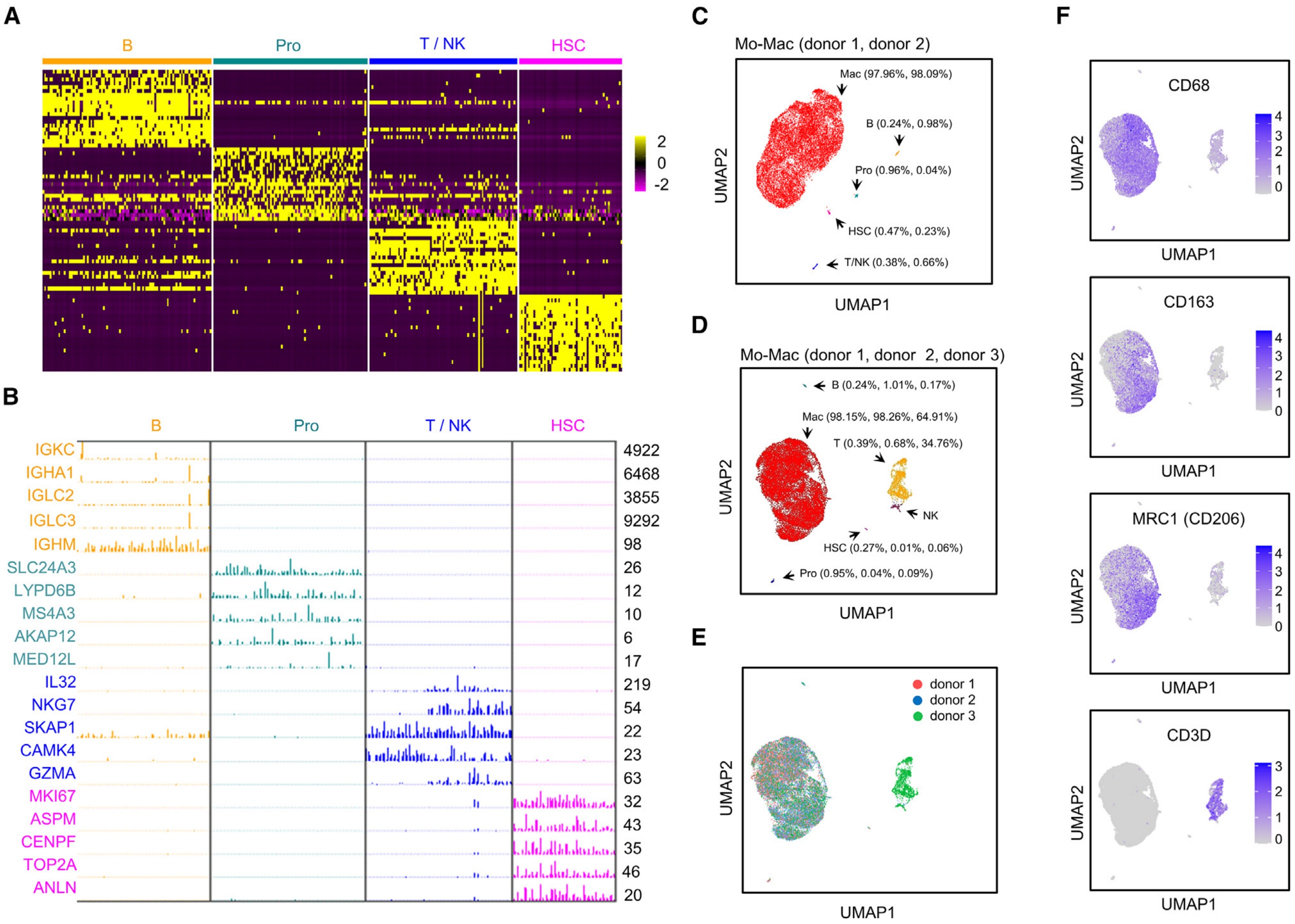

Figure 3. Single-cell transcriptomic analysis of the cell-type composition in Mo-Mac

(A) Heatmap presentation of four non-macrophage types in Mo-Mac, classified by top 20 differentially expressed genes using *Z*-score calculation. B, B cells; Pro, promyelocytes/promonocytes; T, T cells; NK, natural killer cells; HSC, hematopoietic stem cells. (B) Expression levels of marker genes in 323 bystander cells. Top five marker genes for each cell type are presented. Numbers on the right denote maximum detected expression by single-cell analysis. (C) UMAP visualization of five cell types in Mo-Mac manufactured from donor 1 and donor 2 materials (16,301 cells). Numbers in parentheses denote the frequency of indicated cell types from donor 1 and donor 2, respectively. Mac, macrophage. (D) Verification of the single-cell analytical pipeline in (C) using Mo-Mac product manufactured from donor 3 material. Of note, donor 3 was intentionally selected due to excessive presence of lymphocytes in the leukapheresis material. Numbers in parentheses denote the frequency of indicated cell types from donor 1, donor 2, and donor 3, respectively. (E) UMAP visualization of 20,918 cells from three donors after Harmony integration. (F) Expression levels of markers for macrophages (CD68, CD163, CD206) and T cells (CD3D). Side bar denotes relative expression level.

leukapheresis collection is impossible to perform and excessive T cells are present in the final product, macrophages can be further purified with anti-CD14 beads.

Furthermore, we analyzed the expression patterns of anti-inflammatory and proinflammatory markers to determine the cellular state of manufactured macrophages at single-cell resolution. In mice, markers to define the cellular states of macrophages are well established, and the expression pattern of these markers is usually sharply different between unpolarized, M1-polarized, or M2-polarized murine macrophages. However, in humans the situation is more complicated due to variations in genetic background, diets, protocols used by different labs, and other factors. Thus, the markers to define human unpolarized, M1-polarized, or M2-polarized states are not as clear-cut as those in mice. To illustrate this point, we carried out bulk RNA-seq experiments using our manufactured, unpolarized macrophages derived from eight donor materials (donors A–H). We then polarized macrophages from each donor into M1 (induced by IFN-γ) and M2 (induced by IL-4) states, respectively. In total, 24 RNA-seq experiments were performed (M1, unpolarized, and M2 macrophages for each donor), and differential gene expression was analyzed using paired M1 and M2 macrophages from each donor. We selected frequently used markers in the published literature to denote inflammatory state (TNF, IL27, SOCS3, IL23A, CD80, and IL1A) and anti-inflammatory state (MRC1, PPARG, IL1RN, IL10, CCL17, and STAT6). As shown in Figure S1A, inflammatory markers including

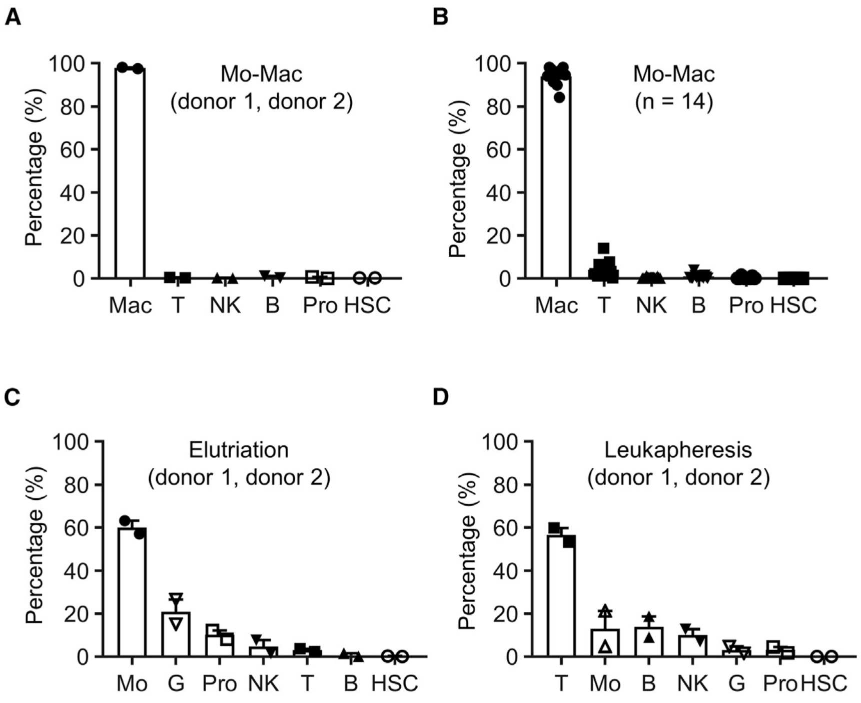

Figure 4. Verification by flow cytometry of cell types identified by single-cell transcriptomic analysis

(A) Frequencies of indicated cell types in Mo-Mac product derived from donor 1 and donor 2. Of note, Mo-Mac product from the same donor was analyzed by single-cell sequencing in Figure 3. Mac, macrophages; T, T cells; NK, natural killer cells; B, B cells; Pro, promyelocytes/promonocytes; HSC, hematopoietic stem cells. (B) Frequencies of indicated cell types in Mo-Mac product derived from an additional 14 donors. T, T cells; NK, natural killer cells; B, B cells; Pro, promyelocytes/promonocytes; HSC, hematopoietic stem cells. (C) Frequencies of indicated cell types in elutriation material derived from donor 1 and donor 2. Mo, monocytes; G, granulocytes; Pro, promyelocytes/promonocytes; NK, natural killer cells; T, T cells; B, B cells; HSC, hematopoietic stem cells. (D) Frequencies of indicated cell types in the starting leukapheresis materials from donor 1 and donor 2. T, T cells; Mo, monocytes; B, B cells; NK, natural killer cells; G, granulocytes; Pro, promyelocytes/promonocytes; HSC, hematopoietic stem cells.

TNF, IL27, SOCS3, and IL23A are higher in M1 compared to paired M2 derived from the same donor. More importantly, the expression level of TNF, IL27, SOCS3, and IL23A in M1 derived from any given donor is higher than M2 derived from all eight donors. In contrast, CD80 expression in M1 from donor A is lower than in M2 from donor F; IL1A expression in M1 from donor E, F, and H is lower than in M2 from donor A. Similarly, regarding anti-inflammatory markers, MRC1, PPARG, and IL1RN are constantly higher in M2 than in M1 macrophages, while other markers are less robust. Thus, we examined the expression levels of TNF, IL27, SOCS3, and IL23A (inflammatory markers) as well as MRC1, PPARG, IL1RN, and IL10 (anti-inflammatory markers) in our single-cell sequencing dataset (Figure S1B). Proinflammatory genes (TNF, IL27, SOCS3, and IL23A) were not significantly expressed. Some anti-inflammatory genes (MRC1, PPARG, and IL1RN) were moderately expressed, but IL10 was minimally expressed. Thus, these results demonstrate that our manufactured macrophages are largely in an unpolarized cellular state.

Verification of Mo-Mac cell-type compositions by flow-cytometry analysis

To verify cell types identified by single-cell transcriptomic analysis for donor 1 and donor 2 (Figure 3), we carried out direct comparison of each cell type between starting material and product using flow cytometry. Gating strategies for macrophages, T cells, B cells, NK cells, HSCs, and promyelocytes/promonocytes in Mo-Mac are shown in Figure S2. In Mo-Mac product manufactured from donor 1 material, the frequencies of immune cells were 98.26% macrophages, 0.35% T cells, 0.00% NK cells, 0.23% B cells, 0.76% promyelocytes/promonocytes, and 0.41% HSCs. For donor 2, the frequencies of immune cells were 97.59% macrophages, 0.49% T cells, 0.30% NK cells, 1.18% B cells, 0.08% promyelocytes/promonocytes, and 0.35% HSCs (Figure 4A). These results demonstrated a high concordance in cell-type composition and frequency between single-cell sequencing and flow-cytometry analysis. Thus, we integrated flow-cytometry analysis of these cell types into quality control procedures. As an example, analyses of Mo-Mac product manufactured from an additional 14 donor materials were conducted. The median frequencies of immune cells were 94.85% macrophages (range 84.18%–98.20%, mean 93.98% ± 1.01%), 3.73% T cells (range 0.28%–13.99%, mean 4.24% ± 0.93%), 0.30% NK cells (range 0.02%–1.12%, mean 0.40% ± 0.08%), 0.77% B cells (range 0.04%–3.94%, mean 1.00% ± 0.25%), 0.13% promyelocytes/promonocytes (range 0.00%–1.66%, mean 0.34% ± 0.13%), and 0.00% HSCs (range 0.00%–0.12%, mean 0.02% ± 0.01%) (Figure 4B).

To trace the origin of these bystander cell types, we analyzed their percentages in elutriation materials. Gating strategies for T cells, B cells, NK cells, monocytes, granulocytes, HSCs, and promyelocytes/promonocytes in elutriation material are shown in Figure S2. The frequencies of immune cells in elutriation material for donor 1 were 63.26% monocytes ($CD14^+$), 15.25% granulocytes ($CD16^+$), 8.17% promyelocytes/promonocytes (negative for lineage cocktail containing CD3/CD14/CD16/CD19/CD20/CD56 and positive for CD13/CD33/HLA-DR), 7.79% NK cells ($CD56^+$), 3.65% T cells ($CD3^+$), 1.58% B cells ($CD19/CD20^+$), and 0.30% HSCs (negative for lineage cocktail containing CD3/CD14/CD16/CD19/CD20/CD56 and positive for CD34). For donor 2, the frequencies were 57.01% monocytes, 26.49% granulocytes, 12.25% promyelocytes/promonocytes, 1.66% NK cells, 2.52% T cells, 0.01% B cells, and 0.07% HSCs (Figure 4C). Interestingly, compared to elutriation material, the percentage of

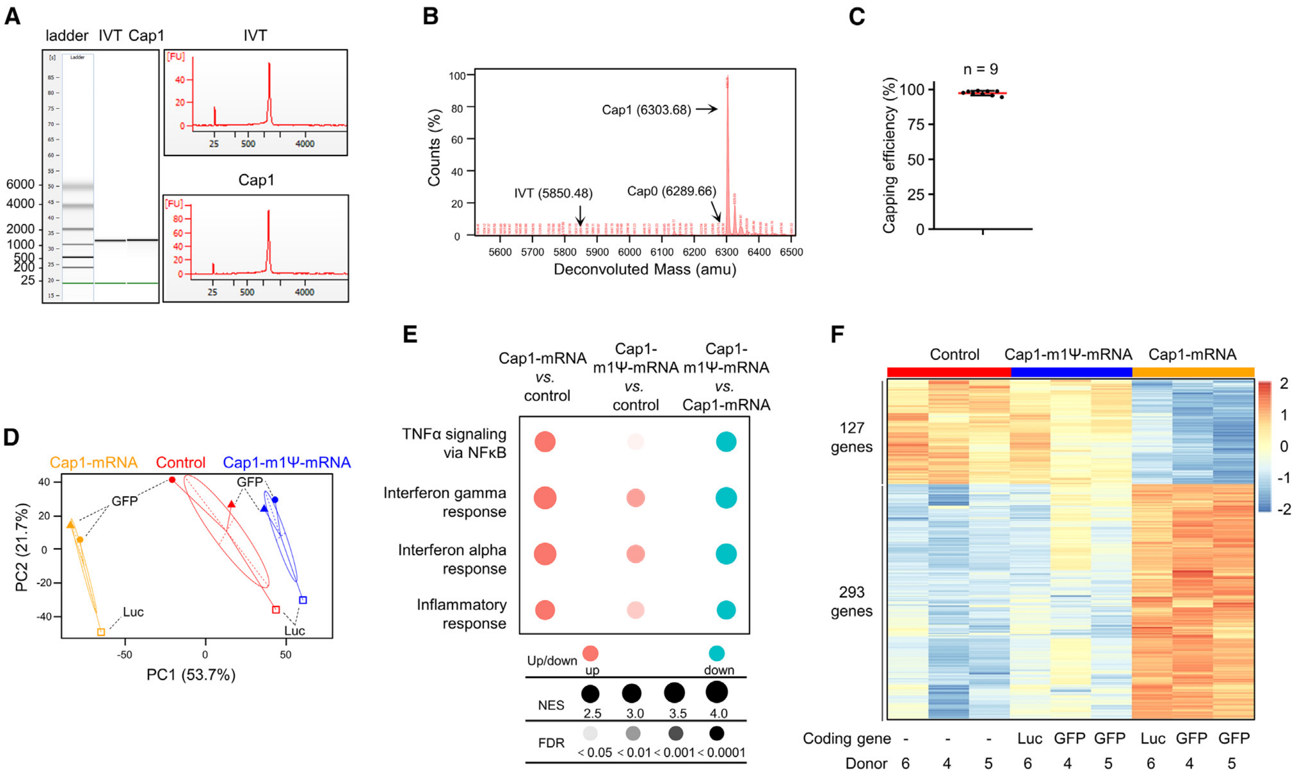

Figure 5. Macrophage engineering with Cap1 and N1-methylpseudouridine modified mRNA

(A) Representative result of *in vitro* transcribed (IVT) RNA. IVT and Cap1 denote IVT RNA without or with a 5′-Cap1 structure (^{m7}GpppGmN), respectively. Gel image (left) and electropherogram (right) by Agilent 2100 Bioanalyzer are shown. (B) Quantification by mass spectrometry of the percentage of Cap1-containing mRNA. Cleavage products of IVT-containing, Cap0 (^{m7}GpppGN)-containing, and Cap1 (^{m7}GpppGmN)-containing RNA are indicated in the deconvoluted mass profile by arrows. (C) Capping efficiencies of nine different mRNA species manufactured under GMP-compliant conditions. The capping efficiency was determined by mass spectrometry as in (B). (D) Principal component (PC) analysis of transcriptomic profiles of macrophages loaded with different mRNAs. Macrophages derived from donor 4 and donor 5 were loaded with Cap1-GFP mRNA or Cap1-m1Ψ-GFP mRNA. Macrophages derived from donor 6 were loaded with Cap1-Luc-mRNA or Cap1-m1Ψ-Luc mRNA. (E) GSEA of differentially expressed pathways in macrophages loaded with Cap1-mRNA or Cap1-m1Ψ-mRNA. (F) Heatmap of the differentially expressed genes in macrophages (control), macrophages loaded with Cap1-mRNA, and macrophages loaded with Cap1-m1Ψ-mRNA.

promyelocytes/promonocytes decreased in Mo-Mac product (Figures 4B and 4C), indicating that promyelocytes/promonocytes might not arise from the culturing process.

We further analyzed the percentage of promyelocytes/promonocytes in leukapheresis materials. The frequency of promyelocytes was 1.81% and 4.60% for donors 1 and 2, respectively (Figure 4D). Because elutriation is a separation method by physical force, it is unlikely that elutriation introduces cell differentiation. Thus, these results demonstrate that promyelocytes/promonocytes originate from leukapheresis and are further enriched by the elutriation process.

Macrophage engineering with 5′-Cap1 and N1-methylpseudouridine-modified mRNA

To expand the functionalities of manufactured macrophages for various therapeutic purposes, we developed a manufacturing process to produce GMP-grade mRNA to express functional proteins of interest. The strong immunogenicity of *in vitro* transcribed (IVT) mRNA primarily originates from uncapped 5′-end and unmodified uridine.[25] Thus, we incorporated IVT mRNA with the Cap1 structure (^{m7}GpppNmN),[26,27] and replaced uridine with N1-methylpseudouridine (m1Ψ).[28,29] The same strategy has been successfully applied in the generation of COVID-19 mRNA vaccines. We were able to generate capped, m1Ψ-modifed mRNAs encoding several potential therapeutic proteins, with various lengths ranging from about 300 to 3,000 nucleotides. NMPA guidelines require the identification of the length integrity and capping efficiency of manufactured mRNA. Here, we used mRNA encoding GFP as an example to demonstrate key quality control steps of mRNA manufacturing. The length integrity of mRNA was routinely monitored by Agilent 2100 using the RNA 6000 Nano Kit (Figure 5A). Note that Cap1-decorated mRNA (Cap1) showed a slightly lower motility than that of uncapped IVT mRNA (Figure 5A). To determine the purity of Cap1-mRNA, liquid chromatography-mass spectrometry was carried out to analyze the 5′ termini of Cap1-mRNA (Figure 5B). A single dominant peak derived from Cap1-mRNA was obvious, with minimal signal peaks indicative

of uncapped IVT mRNA or Cap0-mRNA (Figure 5B). The same analytical procedures were applied for nine mRNAs encoding other proteins. The median capping efficiency was 97.50% (range 94.50%–99.20%, mean 97.38% ± 0.52%) (Figure 5C). In addition, we replaced all uridine with m1Ψ.

To assess the cellular state of mRNA-programmed macrophages, we carried out transcriptomic analysis by RNA-seq. Macrophages derived from donor 4 and donor 5 were electroporated with Cap1-GFP mRNA or Cap1-m1Ψ-GFP mRNA. Typically, there was an approximately 50% loss of cells due to the cytotoxicity of the electroporation procedure. The remaining cells were otherwise highly viable in culture for at least 24 h. To substantiate the RNA-mediated cellular effects (rather than potential effects of GFP protein), Cap1-mRNA encoding luciferase (Luc) was generated without or with m1Ψ modification, Cap1-Luc, and Cap1-m1Ψ-Luc mRNA, respectively. Macrophages derived from donor 6 were electroporated with Cap1-Luc or Cap1-m1Ψ-Luc mRNA. As a control, macrophages were electroporated with water, the solvent used to dissolve manufactured mRNA. Principal component analysis (PCA) clearly distinguished Cap1-GFP/Luc group from the control group and Cap1-m1Ψ-GFP/Luc group (Figure 5D). To functionally verify that our manufactured GMP-grade mRNA was indeed of low immunogenicity, we carried out gene set enrichment analysis (GSEA) using the Hallmark gene set collection in the Molecular Signatures Database (MSigDB).[30] Compared to control, Cap1-GFP as well as Cap1-Luc macrophages significantly upregulated inflammatory pathways including "TNFα signaling via NF-κB," "interferon gamma response" and "interferon alpha response," and "inflammatory response." In contrast, Cap1-m1Ψ-GFP and Cap1-m1Ψ-Luc macrophages exhibited much less activation of these inflammatory pathways (Figure 5E). Lastly, we analyzed differentially expressed genes. To reflect the true effect of RNA and rule out interpersonal variation, only genes induced or repressed in both Cap1-GFP and Cap1-Luc macrophages were included. Again, this analysis showed that differentially expressed genes in Cap1-GFP and Cap1-Luc macrophages were largely attenuated in Cap1-m1Ψ-GFP and Cap1-m1Ψ-Luc macrophages (Figure 5F and Table S1). These results are consistent with the fact that m1Ψ modification on uridine attenuates the immunogenicity of RNA and demonstrate that our macrophage-engineering platform with Cap1-m1Ψ-modifed mRNA can robustly express proteins of interest with minimal immunogenicity (Figure S3).

Macrophages engineered with CD300LF-mRNA facilitate recovery from acute liver damage

We anticipate that mRNA-engineered macrophages can be used in a variety of scenarios to repair tissue damage. Dependent on the underlying pathologies of different diseases, the versatile mRNA technology can provide a solution to encode any therapeutic proteins to enhance the functionalities of macrophages. In the present study, we used drug-induced acute liver damage as a demonstrative case. In humans, overdose of acetaminophen (APAP), also known as paracetamol, is the most common cause of drug-induced liver injury. APAP-induced liver injury may be treatable by antioxidant *N*-acetylcysteine only within a few hours of APAP overdose[31] but otherwise requires liver transplantation.[32] Mouse models of APAP-induced liver damage share the same etiology, and the pathological characteristics resemble those in humans. APAP-induced hepatotoxicity is characterized by excessive cell death and macrophage depletion.[6] We reasoned that enhancing the phagocytic ability of macrophages might help to eliminate necrotic/apoptotic cells to help the regenerative recovery of the liver.

Several genes have been implicated in enhancing the phagocytic ability of macrophages in various biological contexts.[33,34] We generated Cap1-m1Ψ-modifed mRNAs encoding AGER, AXL, CD300LB, and CD300LF. Macrophages derived from nine donors were electroporated with each mRNA individually, and their ability to engulf dead mammalian cells was examined *in vitro*. As a control, macrophages were electroporated with water, the solvent used to dissolve manufactured mRNA. Compared to control, macrophages engineered with Cap1-m1Ψ-CD300LF mRNA exhibited an increased phagocytic ability (Figure 6A) without eliciting inflammatory responses (Figure S4). Thus, we further examined the effect of Cap1-m1Ψ-CD300LF macrophages *in vivo*. In this model, NOD-CB17 Prkdc/SCID mice were injected with APAP to induce acute liver damage and then treated with macrophages (Figure 6B). Cap1-m1Ψ-CD300LF macrophages significantly reduced hepatic cell death (Figure 6C) and lowered the plasma level of alanine transaminase (ALT) and aspartate transaminase (AST) (Figure 6D), indicating a recovery of liver function. Consistently, total necrotic areas, characterized by hepatocyte death and immune cell infiltration, were much smaller in mice treated with Cap1-m1Ψ-CD300LF macrophages (Figure 6E). Taken together, we concluded that macrophages engineered with CD300LF mRNA can facilitate liver recovery from APAP-induced injury.

DISCUSSION

Macrophages are multifunctional immune cells with medical relevance in a variety of human diseases. Their highly malleable nature poses both an opportunity and a challenge for developing cell therapies. In the present study, we demonstrate a GMP-compliant, reproducible manufacturing platform, Mo-Mac, to produce high-quality macrophages. To meet the criteria by regulatory agencies, we used single-cell sequencing to unbiasedly identify cell compositions in Mo-Mac. This analysis also facilitated the development of flow-cytometry-based methodology to routinely monitor the cell compositions in Mo-Mac final product. Furthermore, we established a platform to manufacture mRNA and demonstrated that the functionality of macrophages can be enhanced by versatile mRNA technology. To meet different medical needs, macrophages can be programmed to a variety of functional states by using combinations of various cytokines and mRNA. The present study should provide a framework to guide the development of other GMP-grade macrophage-based cell therapies.

It is critical to carefully choose suitable engineering toolkits to manipulate macrophages for different therapeutic purposes. It is well known

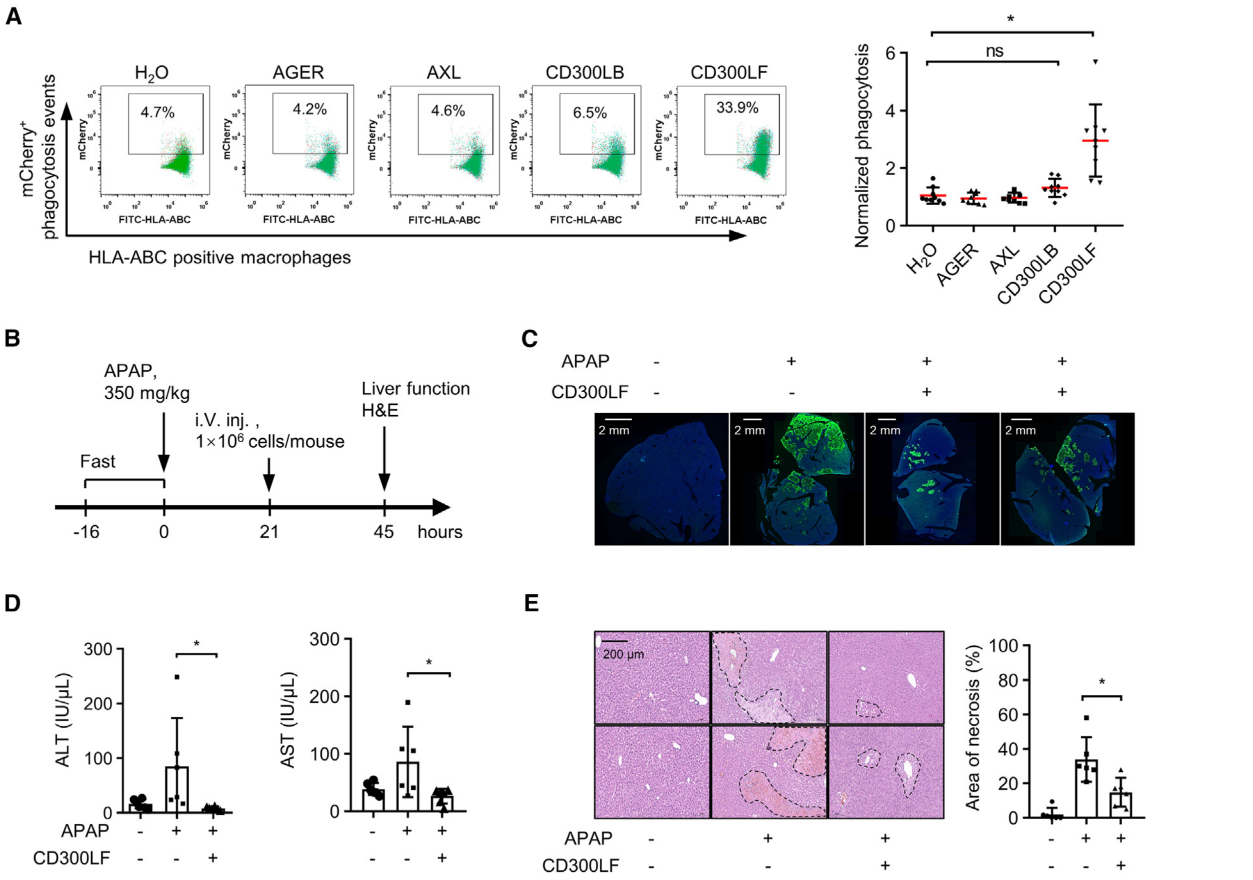

Figure 6. Macrophages engineered with CD300LF-mRNA facilitate recovery from acute liver damage

(A) Phagocytic ability of macrophages electroporated with Cap1-m1Ψ-mRNA encoding indicated genes. Water (H_2O), the dissolvent of Cap1-m1Ψ-mRNA, was used as electroporation control. The ability of mRNA-loaded macrophages to engulf apoptotic cells (labeled with mCherry) were determined by a flow-cytometry-based method and normalized to that of control. The mCherry$^+$ events within the HLA-ABC$^+$ population were plotted as percentage phagocytosis. A representative result is shown (left panel). Macrophages derived from nine donors were loaded with Cap1-m1Ψ-mRNA encoding AGER (gene ID: 177), AXL (gene ID: 558), CD300LB (gene ID: 124599), or CD300LF (gene ID: 146722) individually, and their phagocytic abilities were measured (right panel) (unpaired t test, $p = 0.0004$). (B) Experimental design of using Cap1-m1Ψ-CD300LF mRNA-engineered macrophages to treat acetaminophen (APAP)-induced acute liver damage in mice. (C) Measurement of hepatic cell death by TUNEL assay. (D) Serum alanine aminotransferase (ALT, left panel) and aspartate aminotransferase (AST, right panel) in control mice, APAP-treated mice (APAP), and APAP-treated mice infused with Cap1-m1Ψ-CD300LF mRNA-engineered macrophages (CD300LF). Six mice were used for each group. (E) Representative H&E histological stains of liver sections from control mice, APAP-treated mice, and APAP-treated mice infused with Cap1-m1Ψ-CD300LF mRNA-engineered macrophages (CD300LF). Dotted lines indicate necrotic areas characterized by the infiltration of immune cells and lack of hepatocytes. Areas of necrosis were quantitated for each group (unpaired t test, $p = 0.013$).

that unmodified mRNA incites strong inflammatory responses in macrophages. This proinflammatory reaction would be detrimental if the intended medical application is to repair tissue damage. Consistent with previous studies, we found that Cap1 and m1Ψ modification on mRNA dampened inflammatory responses. Interestingly, our transcriptomic analysis also revealed that unmodified mRNA downregulated a set of genes. How these genes affect macrophage behavior needs further mechanistic investigation. On the other hand, proinflammatory reactions in macrophages would be beneficial in the context of cancer treatment. In this case, unmodified mRNA encoding therapeutic proteins may be desired over m1Ψ-modified mRNA, such as to enhance the proinflammatory M1 phenotype of CAR-M to boost the anti-tumor microenvironment. Lastly, other chemical modifications of RNA are being rapidly developed. It will of interest to examine the biological effects of these novel modifications on macrophages.

Limitations of the study

Macrophage-based therapies to treat human diseases are emerging. The capability to manufacture uniform cells from personalized donor materials is the key to obtaining interpretable clinical results. In this study, we show that the phenotypic markers are relatively uniform in macrophages derived from dozens of healthy donors. It will be essential to evaluate the phenotypic stability and variation of macrophages derived from patient materials. This investigation will be carried out in our clinical studies in the near future. In addition, we show that

macrophages engineered with mRNA encoding CD300LF exhibit enhanced phagocytic capability on dead mammalian cells. It is unclear why other phagocytosis regulators did not work in this context, although they have been shown in mouse studies to increase phagocytosis. One possibility may be species differences.[35] Another may be that these regulators recognize other forms of cell death. Further studies are needed to clarify these issues.

MATERIALS AND METHODS

Monocyte purification by leukapheresis and elutriation

To develop the GMP manufacturing process, peripheral blood mononuclear cells were collected by leukapheresis. Informed consent was obtained from all volunteers for apheresis donation, and the study was approved by the Ethical Committees of West China Second University Hospital at Sichuan University (2019-029). We made a single collection from healthy donors (median age, 33 years; range, 19–56 years) using the Spectra Optia system (Terumo BCT) with mononuclear cell collection procedure. Subsequently, leukapheresis materials were subjected to continuous-counterflow elutriation using the Elutra system (Terumo BCT) in Hanks' balanced salt solution (HBSS; Lonza) supplemented with 1% human albumin. After priming with HBSS, the leukapheresis material was loaded via the inlet pump into the constantly rotating elutriation chamber, using a constant centrifugation speed of 2,400 rpm and increasing the cell medium flow rate step by step (37 mL/min, 97.5 mL/min, 97.5 mL/min, 97.5 mL/min, 103.4 mL/min, and 100 mL/min). The total elutriation time was 1 h. Six fractions were collected, and the percentage of various immune cells was analyzed by an ABX Pentra 60 hematology analyzer (HORIBA). The cellular viability was measured using flow cytometry (Accuri C6 Plus, Becton Dickinson) by staining with propidium iodide and anti-CD45 antibody (BD Biosciences). The percentage of monocytes was determined using flow cytometry (Accuri C6 Plus) by labeling with propidium iodide and anti-CD14 antibody (BD Biosciences). In most circumstances, monocytes were enriched in fraction 4, 5, or 6. Monocytes (1×10^6 cells/mL) were then differentiated into macrophages in gas-permeable plastic bags (MACS GMP Cell Differentiation Bag, Miltenyi). At least 10^9 monocytes were used to manufacture the product. Due to variations in donor materials, monocytes from single fraction or combined fractions were used. Out of 23 batches produced, 16 were manufactured using faction 6 alone, five from combined fractions 5 and 6, and two from combined fractions 4, 5, and 6.

Macrophage manufacture

Monocytes were cultured in gas-permeable plastic bags (Miltenyi) at a density of 1×10^6 cells/mL in TexMACS (Miltenyi) supplemented with 100 ng/mL M-CSF (R&D Systems). Cells were cultured in a humidified atmosphere at 37°C with 5% CO_2 for 7 days. A 50% volume media replenishment was carried out twice during culture (days 2 and 4), with 50% of the culture medium removed and then fed with fresh medium supplemented with 200 ng/mL M-CSF (to restore a final concentration of 100 ng/mL). Macrophages were removed from the culture bags at day 7 using PBS/EDTA buffer containing pharmaceutical grade 0.5% human albumin. Harvested cells were resuspended in CS10 (STEMCELL Technologies) and aliquoted in freezing bags. Each bag was placed in a CryoMed controlled-rate freezer (Thermo Fisher Scientific) until the temperature reached −130°C, then transferred to the gas phase of liquid nitrogen for long-term storage until the day of distribution.

Cell counting and immunophenotyping

The quantity and percentage of various immune cell types in leukapheresis product and elutriation fractions were analyzed by an ABX Pentra 60 hematology analyzer (HORIBA). For other experiments, flow-cytometry analysis (Accuri C6 Plus, Becton Dickinson) was used. Monocytes were enumerated by $CD45^+/CD14^+$ and propidium iodide-negative population (Becton Dickinson). Macrophages were gated by forward scatter and side scatter on the basis of cell size and cellular complexity, and further gated by anti-CD45 and DRAQ7 (live/dead). Phenotypic analyses of macrophages were performed using anti-25F9-APC, CD14-PE, and CD206-FITC (Becton Dickinson). To analyze cell-type compositions in Mo-Mac product, anti-CD45-APC, CD19-PE, CD20-PE, CD3-Percp-Cy5.5, CD56-PE, CD34-PE, lineage cocktail (CD3/CD14/CD16/CD19/CD20/CD56)-FITC, CD13-PE, CD33-PE, and HLA-DR-APC (Becton Dickinson) were used.

Phagocytosis assays

A bioparticles-based phagocytosis assay was routinely carried out as a quality control step to ensure the quality of manufactured macrophages. Macrophages were tested for phagocytic uptake using pHrodo *E. coli* BioParticles (Life Technologies, Thermo Fisher), which fluoresce only when taken into acidic endosomes. In brief, macrophages were cultured with 5 μL of pHrodo *E. coli* BioParticles for 1 h, followed by medium removal and wash with cold PBS. Phagocytosis was then quantified by flow-cytometry analysis. To examine the ability of macrophages to engulf dead mammalian cells, macrophages were electroporated with water or indicated mRNAs and subjected to an apoptotic-cells-based phagocytosis assay. In brief, mCherry-K562 cells were exposed to 2.5 μM shikonin (Selleck) overnight to induce cell death. Macrophages were incubated with shikonin-treated mCherry-K562 cells ($HLA\text{-}ABC^{-/-}$) at a 5:1 ratio for 1 h at 37°C in triplicate. Cell mixtures were then incubated with anti-human HLA-ABC-FITC (BD Biosciences) to label macrophages and analyzed using the BD FACSCelesta cell analyzer. The $mCherry^+$ events within the $HLA\text{-}ABC^+$ population were plotted as percentage phagocytosis.

RNA manufacture

mRNAs encoding AGER, AXL, CD300LB, CD300LF, GFP, and luciferase were transcribed from linearized plasmids using the T7 transcription kit (Novoprotein, China). To produce nucleoside-modified mRNAs, uridine 5′-triphosphate (UTP) was replaced by N1-methyl-pseudouridine (m1ψ, TriLink) in the transcription reaction. To generate 5′-terminal cap structures, vaccinia virus capping enzymes (Novoprotein, China) were used according to the manufacturer's instructions to enzymatically cap the synthesized mRNA with Cap0 or Cap1 structure. The RNA concentration was determined using a

Nanodrop One spectrophotometer (Thermo Fisher Scientific). Aliquots of mRNAs were denatured and analyzed by the Agilent 2100 Bioanalyzer system. To determine the capping efficiency of manufactured mRNA, 5′-mRNA fragments were processed as previously described[36] and analyzed by the Agilent 6545 Q-TOF LC/MS system equipped with an electrospray source operating in negative ionization mode. All mass spectra were obtained in the negative ion mode, over a scan range of m/z 0–3,200. Spectra were analyzed using BioConfirm10.0 software. For mRNA delivery, IVT mRNA was electroporated into macrophages using the Gene Pulser X cell electroporation system (Bio-Rad) following the manufacturer's instruction. After electroporation, cells were cultured for 24 h, and encoded proteins were assessed by corresponding antibodies using flow-cytometry analysis.

Acute liver injury mouse model and macrophage treatment

NOD-CB17 Prkdc/SCID mice were purchased from Vitalstar (Beijing, China) and housed in individually ventilated cages in a sterile animal facility with a 12:12-h dark/light cycle and free access to food and water. All manipulation procedures were performed in accordance with the Institutional Animal Care and Use Committee guideline (S-ACU22-1835). Eight-week-old female mice were fasted for 16 h and received a single acetaminophen (APAP; Selleck) intraperitoneal injection (350 mg/kg). Macrophages (4×10^6 cells/200 μL) were administered intravenously at 21 h after APAP treatment. Mice were humanely culled at 45 h after APAP treatment, and whole blood was collected via cardiac puncture. ALT and AST levels were measured by a commercial kit (Elabscience). Livers were fixed for 48 h at room temperature in 10% neutral-buffered formalin, dehydrated, embedded in paraffin blocks, and sectioned at 7 μm. Sections were stained with H&E and scanned by the Whole Slide Imaging system (Pannoramic MIDI, PerkinElmer). The area of necrosis was quantified by measuring H&E-negative acellular areas (loss of cellular detail) from six randomly selected 5× field images from two sections of each liver using ImageJ. To assess cell death, a TUNEL assay was performed following the manufacturer's instructions (Yeason).

Bioinformatics analyses

For single-cell transcriptomic analysis, cell suspension (20 μL, 10^6/mL, viability >95%) was loaded into chromium microfluidic chips with a 3′ reagent kit (v3.1 chemistry) and barcoded with a 10× Chromium Controller (10× Genomics). Data processing and analyses were carried out using our previously established bioinformatics pipelines.[19] For bulk RNA-seq, a total amount of 1 μg of RNA per sample was used as input material for the RNA sample preparations. In brief, mRNA was purified from total RNA using poly-T oligo-attached magnetic beads. Fragmentation was carried out using divalent cations under elevated temperature in first-strand synthesis reaction buffer (5×). First-strand cDNA was synthesized using random hexamer primer and M-MuLV reverse transcriptase. Second-strand cDNA synthesis was subsequently performed using DNA polymerase I and RNase H. Remaining overhangs were converted into blunt ends via exonuclease/polymerase activities. After adenylation of 3′ ends of DNA fragments, adaptors with hairpin loop structure were ligated to prepare for hybridization. To select cDNA fragments of preferentially 370–420 bp in length, the library fragments were purified with the AMPure XP system (Beckman Coulter, Beverly, MA, USA). PCR was then performed with Phusion high-fidelity DNA polymerase, universal PCR primers, and Index (X) primer. Lastly, PCR products were purified (AMPure XP system), and library quality was assessed on the Agilent Bioanalyzer 2100 system. The library preparations were sequenced on an Illumina Novaseq platform, and 150-bp paired-end reads were generated. The resulting fastq files were quality controlled using FastQC v0.11.8, and adapters were trimmed using Trimmomatic v0.39.[37] Paired-end clean reads were aligned to the reference genome (GRCh38) using Hisat2 v2.0.5.[38] Stringtie was used to count the read numbers mapped to each gene.[39] Transcripts per kilobase of exon model per million mapped reads of each gene were calculated based on the length of the gene, and the reads count was mapped to this gene. To identify differentially expressed genes, the raw count table was created using the DESeq2 R package (1.16.1).[40] The resulting p values were adjusted using the Benjamini-Hochberg approach for conversion to the false discovery rate (FDR). FDR < 0.05 and $|\log_2(\text{fold change})| > 1$ were set as the cutoff for differentially expressed genes. All differentially expressed genes were used to perform PCA with the ade4 v1.7.18 R package. For GSEA, GSEA (v4.1.0) and the Hallmark gene set from MSigDB (https://www.gsea-msigdb.org/) were used.[30] Heatmaps were generated using the pheatmap v1.0.12 R package.

DATA AND CODE AVAILABILITY

All data needed to evaluate the conclusions in the paper are present in the paper. Additional data related to this paper may be requested from the lead corresponding author.

SUPPLEMENTAL INFORMATION

Supplemental information can be found online at https://doi.org/10.1016/j.omtm.2024.101307.

ACKNOWLEDGMENTS

This work was supported by the National Natural Science Foundation of China (31971141, 32271348, 32371539, and 32071284) and the Science and Technology Department of Sichuan Province (2024NSFSC0731).

AUTHOR CONTRIBUTIONS

Conception and design, P.D., J.C., and Q.L.; acquisition of data, P.Z., Y.W., J.J., X.L., T.L., J.Y., and X.M.; analysis and interpretation of data, C.Y., J.C., and Q.L.; writing, review, and/or revision of the manuscript, P.D., J.C., and Q.L.; study supervision, P.D., J.C., and Q.L. All authors reviewed the manuscript.

DECLARATION OF INTERESTS

The authors declare no competing interests.

REFERENCES

1. Park, M.D., Silvin, A., Ginhoux, F., and Merad, M. (2022). Macrophages in health and disease. Cell *185*, 4259–4279. https://doi.org/10.1016/j.cell.2022.10.007.

2. Mass, E., Nimmerjahn, F., Kierdorf, K., and Schlitzer, A. (2023). Tissue-specific macrophages: how they develop and choreograph tissue biology. Nat. Rev. Immunol. *23*, 563–579. https://doi.org/10.1038/s41577-023-00848-y.

3. Epelman, S., Lavine, K.J., and Randolph, G.J. (2014). Origin and functions of tissue macrophages. Immunity *41*, 21–35. https://doi.org/10.1016/j.immuni.2014.06.013.

4. Ginhoux, F., and Guilliams, M. (2016). Tissue-Resident Macrophage Ontogeny and Homeostasis. Immunity *44*, 439–449. https://doi.org/10.1016/j.immuni.2016.02.024.

5. Cox, N., Pokrovskii, M., Vicario, R., and Geissmann, F. (2021). Origins, Biology, and Diseases of Tissue Macrophages. Annu. Rev. Immunol. *39*, 313–344. https://doi.org/10.1146/annurev-immunol-093019-111748.

6. Campana, L., Esser, H., Huch, M., and Forbes, S. (2021). Liver regeneration and inflammation: from fundamental science to clinical applications. Nat. Rev. Mol. Cell Biol. *22*, 608–624. https://doi.org/10.1038/s41580-021-00373-7.

7. Luque-Martin, R., Mander, P.K., Leenen, P.J.M., and Winther, M.P.J. (2021). Classic and new mediators for in vitro modelling of human macrophages. J. Leukoc. Biol. *109*, 549–560. https://doi.org/10.1002/JLB.1RU0620-018R.

8. Bayik, D., Tross, D., Haile, L.A., Verthelyi, D., and Klinman, D.M. (2017). Regulation of the maturation of human monocytes into immunosuppressive macrophages. Blood Adv. *1*, 2510–2519. https://doi.org/10.1182/bloodadvances.2017011221.

9. Murray, P.J. (2017). Macrophage Polarization. Annu. Rev. Physiol. *79*, 541–566. https://doi.org/10.1146/annurev-physiol-022516-034339.

10. Murray, P.J., Allen, J.E., Biswas, S.K., Fisher, E.A., Gilroy, D.W., Goerdt, S., Gordon, S., Hamilton, J.A., Ivashkiv, L.B., Lawrence, T., et al. (2014). Macrophage activation and polarization: nomenclature and experimental guidelines. Immunity *41*, 14–20. https://doi.org/10.1016/j.immuni.2014.06.008.

11. Cassetta, L., and Pollard, J.W. (2023). A timeline of tumour-associated macrophage biology. Nat. Rev. Cancer *23*, 238–257. https://doi.org/10.1038/s41568-022-00547-1.

12. Mantovani, A., Allavena, P., Marchesi, F., and Garlanda, C. (2022). Macrophages as tools and targets in cancer therapy. Nat. Rev. Drug Discov. *21*, 799–820. https://doi.org/10.1038/s41573-022-00520-5.

13. Barry, S.T., Gabrilovich, D.I., Sansom, O.J., Campbell, A.D., and Morton, J.P. (2023). Therapeutic targeting of tumour myeloid cells. Nat. Rev. Cancer *23*, 216–237. https://doi.org/10.1038/s41568-022-00546-2.

14. Christofides, A., Strauss, L., Yeo, A., Cao, C., Charest, A., and Boussiotis, V.A. (2022). The complex role of tumor-infiltrating macrophages. Nat. Immunol. *23*, 1148–1156. https://doi.org/10.1038/s41590-022-01267-2.

15. Na, Y.R., Kim, S.W., and Seok, S.H. (2023). A new era of macrophage-based cell therapy. Exp. Mol. Med. *55*, 1945–1954. https://doi.org/10.1038/s12276-023-01068-z.

16. Klichinsky, M., Ruella, M., Shestova, O., Lu, X.M., Best, A., Zeeman, M., Schmierer, M., Gabrusiewicz, K., Anderson, N.R., Petty, N.E., et al. (2020). Human chimeric antigen receptor macrophages for cancer immunotherapy. Nat. Biotechnol. *38*, 947–953. https://doi.org/10.1038/s41587-020-0462-y.

17. Sawitzki, B., Harden, P.N., Reinke, P., Moreau, A., Hutchinson, J.A., Game, D.S., Tang, Q., Guinan, E.C., Battaglia, M., Burlingham, W.J., et al. (2020). Regulatory cell therapy in kidney transplantation (The ONE Study): a harmonised design and analysis of seven non-randomised, single-arm, phase 1/2A trials. Lancet *395*, 1627–1639. https://doi.org/10.1016/S0140-6736(20)30167-7.

18. Fontaine, M.J., Selogie, E., Stroncek, D., McKenna, D., Szczepiorkowski, Z.M., Takanashi, M., Garritsen, H., Girdlestone, J., and Reems, J.A.; Biomedical Excellence for Safer Transfusion BEST Collaborative (2020). Variations in novel cellular therapy products manufacturing. Cytotherapy *22*, 337–342. https://doi.org/10.1016/j.jcyt.2020.01.012.

19. Li, Q., Yang, C., Tian, H., Jiang, J., Li, P., Zhu, X., Lei, T., Yin, R., Ding, P., Bai, P., and Li, Q. (2023). Development of a personalized dendritic cell vaccine and single-cell RNA sequencing-guided assessment of its cell type composition. Cytotherapy *25*, 210–219. https://doi.org/10.1016/j.jcyt.2022.10.013.

20. Zwadlo, G., Bröcker, E.B., von Bassewitz, D.B., Feige, U., and Sorg, C. (1985). A monoclonal antibody to a differentiation antigen present on mature human macrophages and absent from monocytes. J. Immunol. *134*, 1487–1492.

21. Korsunsky, I., Millard, N., Fan, J., Slowikowski, K., Zhang, F., Wei, K., Baglaenko, Y., Brenner, M., Loh, P.R., and Raychaudhuri, S. (2019). Fast, sensitive and accurate integration of single-cell data with Harmony. Nat. Methods *16*, 1289–1296. https://doi.org/10.1038/s41592-019-0619-0.

22. Stuart, T., Butler, A., Hoffman, P., Hafemeister, C., Papalexi, E., Mauck, W.M., 3rd, Hao, Y., Stoeckius, M., Smibert, P., and Satija, R. (2019). Comprehensive Integration of Single-Cell Data. Cell *177*, 1888–1902. https://doi.org/10.1016/j.cell.2019.05.031.

23. Aran, D., Looney, A.P., Liu, L., Wu, E., Fong, V., Hsu, A., Chak, S., Naikawadi, R.P., Wolters, P.J., Abate, A.R., et al. (2019). Reference-based analysis of lung single-cell sequencing reveals a transitional profibrotic macrophage. Nat. Immunol. *20*, 163–172. https://doi.org/10.1038/s41590-018-0276-y.

24. Hao, Y., Hao, S., Andersen-Nissen, E., Mauck, W.M., 3rd, Zheng, S., Butler, A., Lee, M.J., Wilk, A.J., Darby, C., Zager, M., et al. (2021). Integrated analysis of multimodal single-cell data. Cell *184*, 3573–3587. https://doi.org/10.1016/j.cell.2021.04.048.

25. Kariko, K., Buckstein, M., Ni, H., and Weissman, D. (2005). Suppression of RNA recognition by Toll-like receptors: the impact of nucleoside modification and the evolutionary origin of RNA. Immunity *23*, 165–175. https://doi.org/10.1016/j.immuni.2005.06.008.

26. Devarkar, S.C., Wang, C., Miller, M.T., Ramanathan, A., Jiang, F., Khan, A.G., Patel, S.S., and Marcotrigiano, J. (2016). Structural basis for m7G recognition and 2'-O-methyl discrimination in capped RNAs by the innate immune receptor RIG-I. Proc. Natl. Acad. Sci. USA *113*, 596–601. https://doi.org/10.1073/pnas.1515152113.

27. Schuberth-Wagner, C., Ludwig, J., Bruder, A.K., Herzner, A.M., Zillinger, T., Goldeck, M., Schmidt, T., Schmid-Burgk, J.L., Kerber, R., Wolter, S., et al. (2015). A Conserved Histidine in the RNA Sensor RIG-I Controls Immune Tolerance to N1-2'O-Methylated Self RNA. Immunity *43*, 41–51. https://doi.org/10.1016/j.immuni.2015.06.015.

28. Andries, O., Mc Cafferty, S., De Smedt, S.C., Weiss, R., Sanders, N.N., and Kitada, T. (2015). N(1)-methylpseudouridine-incorporated mRNA outperforms pseudouridine-incorporated mRNA by providing enhanced protein expression and reduced immunogenicity in mammalian cell lines and mice. J. Control. Release *217*, 337–344. https://doi.org/10.1016/j.jconrel.2015.08.051.

29. Kim, K.Q., Burgute, B.D., Tzeng, S.C., Jing, C., Jungers, C., Zhang, J., Yan, L.L., Vierstra, R.D., Djuranovic, S., Evans, B.S., and Zaher, H.S. (2022). N1-methylpseudouridine found within COVID-19 mRNA vaccines produces faithful protein products. Cell Rep. *40*, 111300. https://doi.org/10.1016/j.celrep.2022.111300.

30. Liberzon, A., Birger, C., Thorvaldsdóttir, H., Ghandi, M., Mesirov, J.P., and Tamayo, P. (2015). The Molecular Signatures Database (MSigDB) hallmark gene set collection. Cell Syst. *1*, 417–425. https://doi.org/10.1016/j.cels.2015.12.004.

31. Licata, A., Minissale, M.G., Stankevičiūtė, S., Sanabria-Cabrera, J., Lucena, M.I., Andrade, R.J., and Almasio, P.L. (2022). N-Acetylcysteine for Preventing Acetaminophen-Induced Liver Injury: A Comprehensive Review. Front. Pharmacol. *13*, 828565. https://doi.org/10.3389/fphar.2022.828565.

32. Chiew, A.L., Gluud, C., Brok, J., and Buckley, N.A. (2018). Interventions for paracetamol (acetaminophen) overdose. Cochrane Database Syst. Rev. *2*, CD003328. https://doi.org/10.1002/14651858.CD003328.pub3.

33. Lemke, G. (2019). How macrophages deal with death. Nat. Rev. Immunol. *19*, 539–549. https://doi.org/10.1038/s41577-019-0167-y.

34. Boada-Romero, E., Martinez, J., Heckmann, B.L., and Green, D.R. (2020). The clearance of dead cells by efferocytosis. Nat. Rev. Mol. Cell Biol. *21*, 398–414. https://doi.org/10.1038/s41580-020-0232-1.

35. Monnier, M., Paolini, L., Vinatier, E., Mantovani, A., Delneste, Y., and Jeannin, P. (2022). Antitumor strategies targeting macrophages: the importance of considering the differences in differentiation/polarization processes between human and mouse macrophages. J. Immunother. Cancer *10*, e005560. https://doi.org/10.1136/jitc-2022-005560.

36. Beverly, M., Dell, A., Parmar, P., and Houghton, L. (2016). Label-free analysis of mRNA capping efficiency using RNase H probes and LC-MS. Anal. Bioanal. Chem. *408*, 5021–5030. https://doi.org/10.1007/s00216-016-9605-x.

37. Bolger, A.M., Lohse, M., and Usadel, B. (2014). Trimmomatic: a flexible trimmer for Illumina sequence data. Bioinformatics *30*, 2114–2120. https://doi.org/10.1093/bioinformatics/btu170.

38. Kim, D., Paggi, J.M., Park, C., Bennett, C., and Salzberg, S.L. (2019). Graph-based genome alignment and genotyping with HISAT2 and HISAT-genotype. Nat. Biotechnol. *37*, 907–915. https://doi.org/10.1038/s41587-019-0201-4.

39. Pertea, M., Pertea, G.M., Antonescu, C.M., Chang, T.C., Mendell, J.T., and Salzberg, S.L. (2015). StringTie enables improved reconstruction of a transcriptome from RNA-seq reads. Nat. Biotechnol. *33*, 290–295. https://doi.org/10.1038/nbt.3122.

40. Love, M.I., Huber, W., and Anders, S. (2014). Moderated estimation of fold change and dispersion for RNA-seq data with DESeq2. Genome Biol. *15*, 550. https://doi.org/10.1186/s13059-014-0550-8.

OMTM, Volume *32*

Supplemental information

Macrophage manufacturing and engineering with 5′-Cap1 and N1-methylpseudouridine-modified mRNA

Peixuan Zhang, Yantai Wang, Jinfeng Jiang, Chao Yang, Xianxia Liu, Tingjun Lei, Xiangjun Meng, Jihong Yang, Ping Ding, Jie Chen, and Qintong Li

A

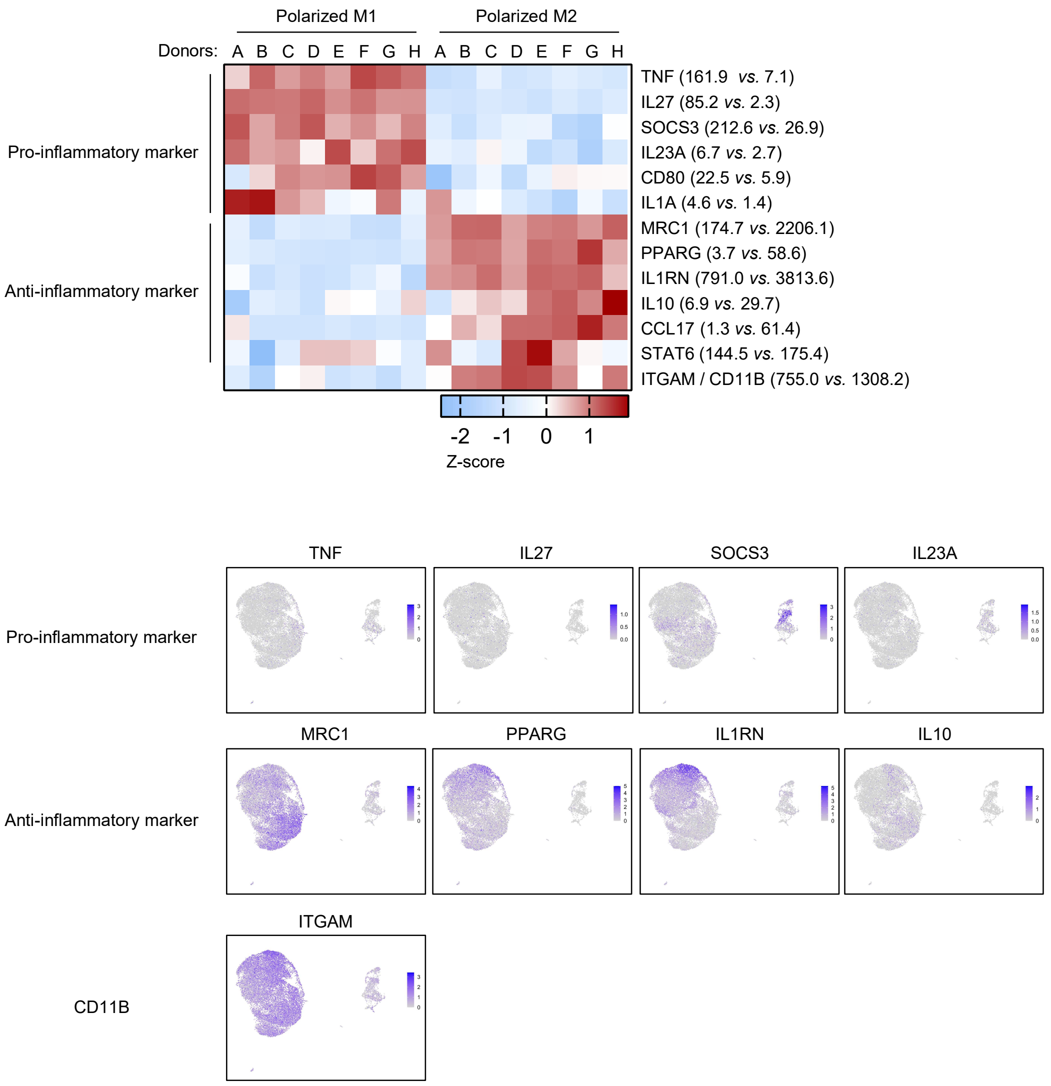

Figure S1. Selection of anti-inflammatory and pro-inflammatory marker genes by comparing paired unpolarized, M1 and M2 macrophages, and their expression pattern at single-cell resolution

(A) Heatmap presentation of inter-personal variation of frequently used inflammatory (TNF, IL27, SOCS3, IL23A, CD80 and IL1A) and anti-inflammatory (MRC1, PPARG, IL1RN, IL10, CCL17 and STAT6) markers in the published literature. Unpolarized macrophages were derived from 8 donor materials (Donors A-H), and polarized individually into M1 (induced by IFN-γ) and M2 state (induced by IL-4), respectively. In total, 24 RNA-seq experiments were performed (M1, unpolarized and M2 for each donor), and differential gene expression was analyzed using paired M1 and M2 macrophages from each donor. Of note, only TNF, IL27, SOCS3 and IL23A (but not CD80 or IL1A) are expressed at higher levels in M1 derived from any given donor than M2 derived from all 8 donors. Similarly, regarding anti-inflammatory markers, MRC1, PPARG and IL1RN are constantly higher in M2 than M1, while other markers are less robust.

(B) UMAP visualization of the expression pattern of TNF, IL27, SOCS3 as well as IL23A (inflammatory markers), and MRC1, PPARG, IL1RN and IL10 (anti-inflammatory markers) as well as CD11B in our single-cell sequencing dataset (Figure 3).

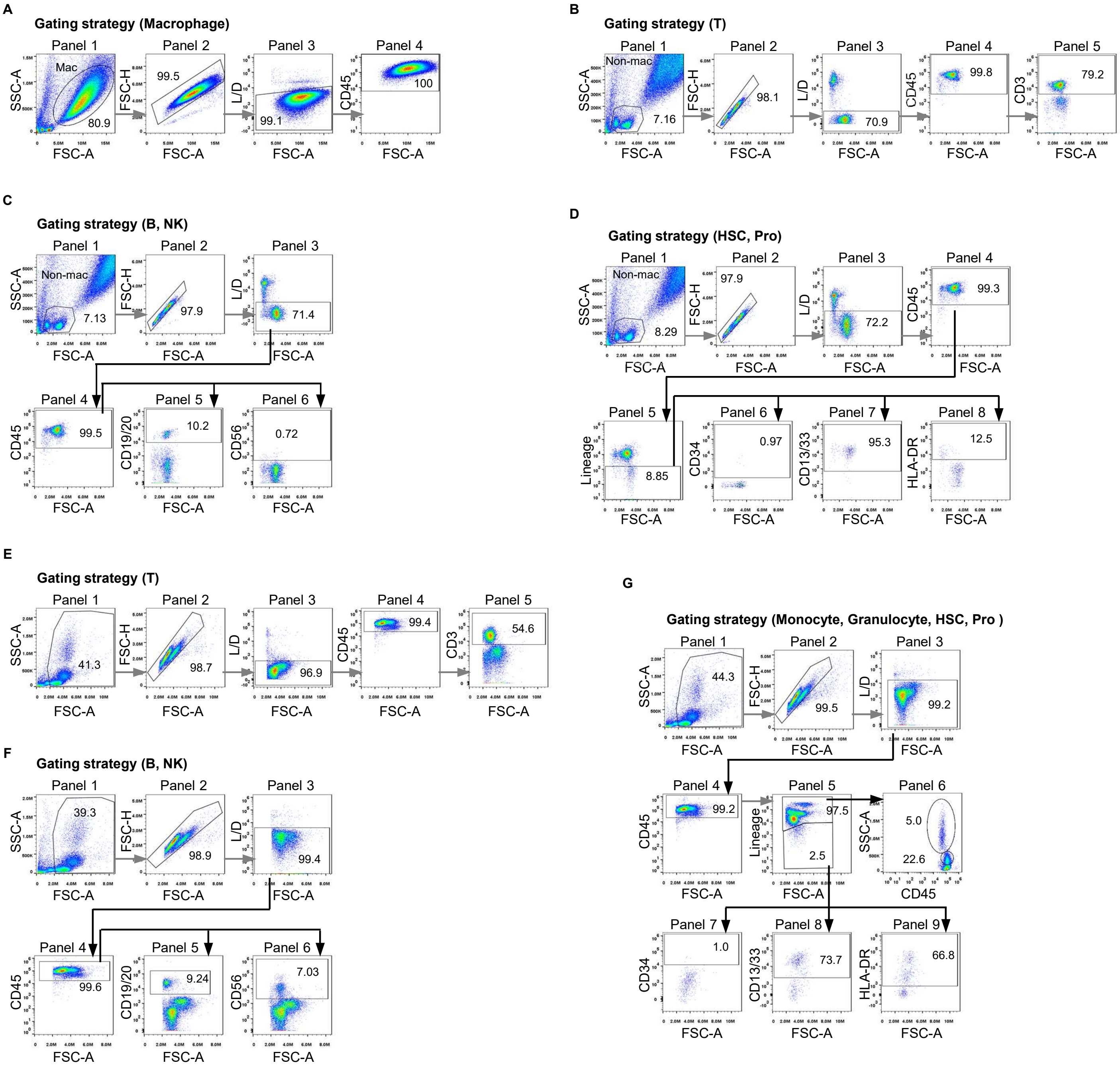
A
Gating strategy (Macrophage)
Panel 1
Panel 2
Panel 3
Panel 4
Mac
80.9
99.5
99.1
100
SSC-A
FSC-H
L/D
CD45
FSC-A
B
Gating strategy (T)
Non-mac
7.16
98.1
70.9
99.8
79.2
CD3
C
Gating strategy (B, NK)
7.13
97.9
71.4
99.5
10.2
0.72
CD19/20
CD56
D
Gating strategy (HSC, Pro)
8.29
97.9
72.2
99.3
8.85
0.97
95.3
12.5
Lineage
CD34
CD13/33
HLA-DR
E
Gating strategy (T)
41.3
98.7
96.9
99.4
54.6
F
Gating strategy (B, NK)
39.3
98.9
99.4
99.6
9.24
7.03
G
Gating strategy (Monocyte, Granulocyte, HSC, Pro)
44.3
99.5
99.2
99.2
97.5
2.5
5.0
22.6
1.0
73.7
66.8
Panel 5
Panel 6
Panel 7
Panel 8
Panel 9

Figure S2. Gating strategies for various cell types

(A) Gating strategy of FACS analyses to quantify macrophages in Mo-Mac. Cells were gated based on side scatter area (SSC-A) and forward scatter area (FSC-A) (panel 1), then gated sequentially to include singlet cells (panel 2), live cells (panel 3) and CD45^{+} cells (panel 4).

(B) Gating strategy to quantitate T cells in Mo-Mac. Non-macrophage cells (Non-mac) were gated based on side scatter area (SSC-A) and forward scatter area (FSC-A) (panel 1), then sequentially gated to include singlet cells (panel 2), live cells (panel 3) and CD45^{+} cells (panel 4). CD45^{+} cells were further gated to identify CD3^{+} T cells (panel 5).

(C) Gating strategy to quantify B and natural killer (NK) cells in Mo-Mac. Non-macrophage cells (Non-mac) were gated based on side scatter area (SSC-A) and forward scatter area (FSC-A) (panel 1), then sequentially gated to include singlet cells (panel 2), live cells (panel 3) and CD45^{+} cells (panel 4). CD45^{+} cells were further gated to identify CD19^{+}CD20^{+} B cells (panel 5) and CD56^{+} NK cells (panel 6).

(D) Gating strategy to quantify hemopoietic stem cells (HSC) and promyelocytes/promonocytes (Pro) in Mo-Mac. Non-macrophage cells (Non-mac) were gated based on side scatter area (SSC-A) and forward scatter area (FSC-A) (panel 1), then sequentially gated to include singlet cells (panel 2), live cells (panel 3) and CD45^{+} cells (panel 4). CD45^{+} cells were gated for lineage-negative cells (CD3^{-}, CD16^{-}, CD19^{-}, CD20^{-}, CD56^{-} and CD14^{-}) (panel 5). Lineage-negative cells were then gated to identify CD34^{+} HSC (panel 6), and CD13^{+}CD33^{+} promyelocytes or HLA-DR^{+} promonocytes (panel 7 and 8).

(E) Gating strategy to quantify T cells in elutriation and leukapheresis materials. Cells were gated based on side scatter area (SSC-A) and forward scatter area (FSC-A) (panel 1), then gated sequentially to include singlet cells (panel 2), live cells (panel 3) and CD45^{+} cells (panel 4). CD45^{+} cells were further gated to identify CD3^{+} T cells (panel 5).

(F) Gating strategy to quantify B and natural killer (NK) cells in elutriation and leukapheresis materials. Cells were gated based on side scatter area (SSC-A) and forward scatter area (FSC-A) (panel 1), then gated sequentially to include singlet cells (panel 2), live cells (panel 3) and CD45^{+} cells (panel 4). CD45^{+} cells were further gated to identify CD19^{+}CD20^{+} B cells (panel 5) and CD56^{+} NK cells (panel 6).

(G) Gating strategy to quantify monocytes, granulocytes, hematopoietic stem cells (HSC) and promyelocytes/promonocytes (Pro) in elutriation and leukapheresis materials. Cells were gated based on side scatter area (SSC-A) and forward scatter area (FSC-A) (panel 1), then gated sequentially to include singlet cells (panel 2), live cells (panel 3) and CD45^{+} cells (panel 4). CD45^{+} cells were gated for lineage-negative cells (CD3^{-}, CD16^{-}, CD19^{-}, CD20^{-}, CD56^{-} and CD14^{-}) (panel 5). Lineage-negative cells were then gated to identify CD34^{+} HSC (panel 7), and CD13^{+}CD33^{+} promyelocytes or HLA-DR^{+} promonocytes (panel 8 and 9). Lineage-positive cells were then gated to identify CD45^{low} granulocyte (panel 6) and CD45^{high} monocyte (panel 6).

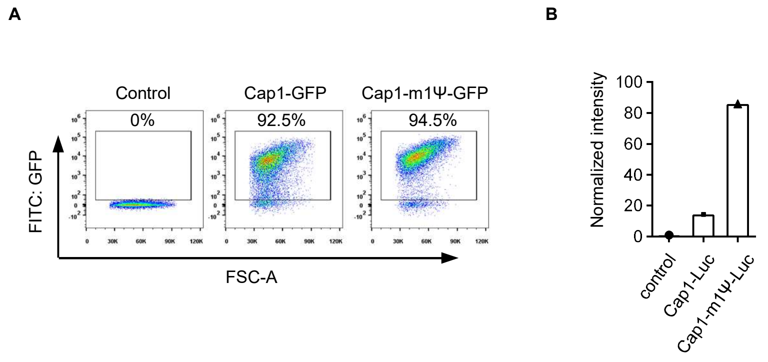

Figure S3. Verification of the expression of Cap1 and Cap1-m1Ψ mRNA

(A) Flow cytometry analysis of the expression of Cap1-GFP mRNA and Cap1-m1Ψ-GFP mRNA in electroporated macrophages.

(B) Bioluminescence-based detection of the expression of Cap1-Luc mRNA and Cap1-m1Ψ-Luc mRNA in electroporated macrophages.

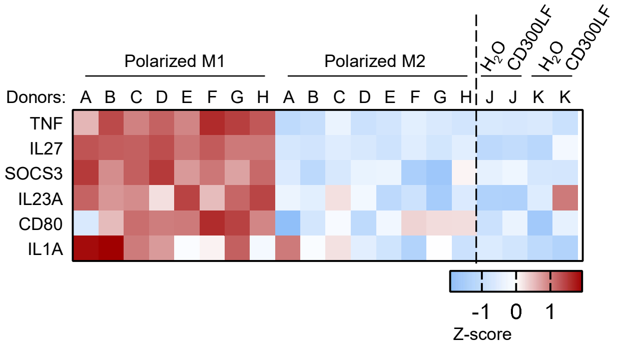

Figure S4. Cap1-m1Ψ-CD300LF mRNA-engineered macrophages are not in an inflammatory state

Heatmap showing the expression level of pro-inflammatory genes (TNF, IL27, SOCS3, IL23A) in Cap1-m1Ψ-CD300LF mRNA-engineered macrophages, M1- or M2-polarized macrophages. Considering the intra-personal variations, only constantly upregulated genes in M1-macrophages were selected as pro-inflammatory markers (Figure S1).

Table S1. Top20 differentially expressed genes induced by unmodified mRNA and attenuated by m1Ψ-modified mRNA

gene_name	Upregulated by unmodified mRNA （fold change）		gene_name	Downregulated by unmodified mRNA （fold change）	
	Cap1 mRNA vs. Control	Cap1-m1Ψ mRNA vs. Control		Cap1 mRNA vs. Control	Cap1-m1Ψ mRNA vs. Control
CXCL11	1002.5	12.4	JAML	-32.8	-1.6
CXCL10	922.3	10.8	FABP4	-22.7	-1.3
ACOD1	540.3	91.7	LINC01503	-22.1	-2.1
IDO1	519.0	44.0	AL844908.1	-19.7	-1.3
CCL8	420.0	9.0	AC078850.1	-18.1	-1.0
AC007991.2	367.2	26.2	DOK2	-17.3	-1.3
APOBEC3A	282.3	20.1	SERPINE1	-14.7	-1.2
TNFAIP6	263.2	4.9	MACROD1	-14.6	-1.7
CD38	210.7	11.0	AL031123.1	-14.5	-1.7
CALHM6	203.1	9.5	SNAI3	-14.3	-2.4
GBP1P1	163.4	15.8	LPL	-13.9	-2.0
PDGFRL	135.5	9.5	LINC01010	-13.7	-1.5
GJA4	134.9	31.8	DBP	-13.3	-1.5
IL27	126.2	1.8	RASAL1	-12.4	-1.5
NEURL3	119.9	5.1	SIGLEC7	-12.0	-1.5
IGFBP4	107.9	4.6	ASGR1	-12.0	-1.5
IFITM1	103.3	11.3	CD300LB	-11.5	-1.2
ZBP1	90.0	6.1	PC	-10.9	-1.4
TNFSF10	85.6	7.0	TM4SF19	-10.8	-1.8
LAG3	82.9	7.9	DPCD	-10.7	-1.4
